# Genomic and transcriptomic insights into the ecology and metabolism of benthic archaeal cosmopolitan, Thermoprofundales (MBG-D archaea)

**DOI:** 10.1038/s41396-018-0321-8

**Published:** 2018-12-04

**Authors:** Zhichao Zhou, Yang Liu, Karen G. Lloyd, Jie Pan, Yuchun Yang, Ji-Dong Gu, Meng Li

**Affiliations:** 1 Institute for Advanced Study, Shenzhen University, 518060 Shenzhen, People’s Republic of China; 2 Department of Microbiology, University of Tennessee, Knoxville, TN 37996 USA; 3 Laboratory of Environmental Microbiology and Toxicology, School of Biological Sciences, The University of Hong Kong, Pokfulam Road, Hong Kong SAR, Hong Kong, People’s Republic of China

**Keywords:** Metagenomics, Microbial ecology

## Abstract

Marine Benthic Group D (MBG-D) archaea, discovered by 16S rRNA gene survey decades ago, are ecologically important, yet understudied and uncultured sedimentary archaea. In this study, a comprehensive meta-analysis based on the 16S rRNA genes of MBG-D archaea showed that MBG-D archaea are one of the most frequently found archaeal lineages in global sediment with widespread distribution and high abundance, including 16 subgroups in total. Interestingly, some subgroups show significant segregations toward salinity and methane seeps. Co-occurrence analyses indicate significant non-random association of MBG-D archaea with Lokiarchaeota (in both saline and freshwater sediments) and Hadesarchaea, suggesting potential interactions among these archaeal groups. Meanwhile, based on four nearly complete metagenome-assembled genomes (MAGs) and corresponding metatranscriptomes reconstructed from mangrove and intertidal mudflat sediments, we provide insights on metabolic potentials and ecological functions of MBG-D archaea. MBG-D archaea appear to be capable of transporting and assimilating peptides and generating acetate and ethanol through fermentation. Metatranscriptomic analysis suggests high expression of genes for acetate and amino acid utilization and for peptidases, especially the M09B-type extracellular peptidase (collagenase) showing high expression levels in all four mangrove MAGs. Beyond heterotrophic central carbon metabolism, the MBG-D genomes include genes that might encode two autotrophic pathways: Wood–Ljundahl (WL) pathways using both H_4_MPT and H_4_folate as C_1_ carriers, and an incomplete dicarboxylate/4-hydroxybutyrate cycle with alternative bypasses from pyruvate to malate/oxaloacetate during dicarboxylation. These findings reveal MBG-D archaea as an important ubiquitous benthic sedimentary archaeal group with specific mixotrophic metabolisms, so we proposed the name Thermoprofundales as a new Order within the Class Thermoplasmata. Globally, Thermoprofundales and other benthic archaea might synergistically transform benthic organic matter, possibly playing a vital role in sedimentary carbon cycle.

## Introduction

Archaea in the subsurface ecosystem play crucial roles in global biogeochemical cycles. The estimated global subsurface sedimentary microbial abundance reaches 2.9 × 10^29^, comprising around 9.1–31.5% of the total number of prokaryotes on the Earth [1]. Recent studies highlighted the vast deposit of archaeal cellular biomass in marine subsurface sediments buried to a depth of >1 m in a wide range of oceanographic settings [2]. Cell membrane lipid studies also show evidence of more living archaea than bacteria [2] and archaea possessing the active metabolic capacity to assimilate sedimentary organic compounds [3]. Within these subsurface environmental settings, the general archaeal cosmopolitans, such as Marine Benthic Group B (MBG-B), Marine Benthic Group D (MBG-D), and Bathyarchaeota, are dominant archaeal groups, which might contribute significantly to biogeochemical cycles [3–6]. MBG-D archaea have long been recognized from 16S rRNA gene surveys in benthic environments, and their global distribution and abundance place them as universal players in both terrestrial and marine subsurface realms (Supplementary Table S1) [5, 7–9]. DNA-based 16S rRNA gene community analyses suggest that MBG-D archaea have specific environmental niches and co-occurrence patterns. They co-occur with anaerobic methanotrophic archaea in methane-driven seeps [10], are abundant in liquid CO_2_ or CO_2_ hydrate-bearing marine sediments [10], and their 16S rRNA gene abundance appears to be independent of biogeochemical zones of sulfate reduction and methanogenesis [2, 5, 11]. Furthermore, MBG-D archaea are also found to progressively replace methanogens going downcore in samples from a freshwater lake [12], and they are also abundant and persistent in hypersaline environments and exhibit small variations of community composition correlated with the change of carbon content [13].

In recent years, genome contents and metabolic pathways of MBG-D archaea have been explored using single-cell genomic and metagenomic approaches [5, 14]. MBG-D archaea are thought to be benthic anaerobic archaea capable of exogenous protein mineralization and acetogenesis [5, 14]. They could secrete active extracellular peptidases in marine sediments [5]. Furthermore, a metagenomic survey reveals that MBG-D archaea and Bathyarchaeota co-exist in White Oak River estuary sediments with high abundance, sharing similar inferred metabolic capacities for acetogenesis and protein degradation in estuarine organic-rich regimes [14, 15]. Owing to their potential importance in carbon transformation and ubiquitous distribution, it is important to have a broader view of the ecological, genomic, and metabolic understanding of MBG-D archaea. However, the few available partial genomes limit our thorough understanding of their global ecological roles and metabolisms. The ecological diversity, genomic blueprints, metabolic properties, and biogeochemical functions of MBG-D archaea remain elusive, though MBG-D archaea have been identified for many years.

Here we conducted a comprehensive meta-analysis based on the available 16S rRNA gene sequences of MBG-D archaea to investigate their global environmental distribution, the environmental associations of different subgroups, and the potential synergistic relationship with other archaeal lineages. We also resolved four nearly complete MBG-D metagenome-assembled genomes (MAGs) from subsurface sediments of mangrove forests and intertidal mudflats in Mai Po Nature Reserve, Hong Kong and one additional MBG-D MAG from the publicly available dataset. These MAGs, together with their metatranscriptomes (Mai Po), provided a better insight on the active metabolic and ecological functions of MBG-D archaea. Based on the unique phylogenetic position and metabolic potentials, we proposed MBG-D archaea as a new order Thermoprofundales within the class Thermoplasmata. Finally, we also addressed the relationships of distribution patterns to metabolic capacities and proposed the potential biogeochemical roles of these ubiquitous sedimentary archaea in carbon cycling.

## Materials and methods

### Sampling, nucleic acids extraction, and metagenome/metatranscriptome sequencing

Sediment samples for DNA extraction were collected from Mai Po Nature Reserve on September 12, 2014. Mai Po Nature Reserve is characterized as a subtropical, coastal wetland with a variety of wetland types, such as intertidal mudflats, mangrove forest, shrimp ponds, and manmade fishery ponds [16]. One subsurface sediment sample (MaiPo-8) was collected from a site covered by mangrove forest (22°29.875′N, 114°01.767′E) at a sediment depth of 10–15 cm, and a deeper sediment sample (MaiPo-9, at 20–25 cm depth) was also collected at the same site. Another sediment sample (MaiPo-11) was collected from a nearby intertidal mudflat site (22°29.949′N, 114°01.656′E) at a depth of 13–16 cm, which was a more homogeneous fine slurry with more reduced redox state than the former two sediment samples. The detailed sampling descriptions and physicochemical parameters are listed in Supplementary Information Note 1 as well as in our previous studies [17, 18]. Bulk sediment DNA (10 g) was isolated according to the manufacturer’s instructions (DNeasy PowerMax Soil Kit, QIAGEN) and concentrated for metagenome sequencing (Novogene Inc., Beijing, China). The samples for metatranscriptomic analysis were also sampled from the same sites and layers as those used for metagenomes at a later time (details in Supplementary Information Note 1). The sediment samples were preserved immediately after sampling with the LifeGuard Soil Preservation Solution (QIAGEN) to prevent RNA degradation. Total RNA was isolated from bulk sediments (5–25 g) according to the manufacturer’s instructions (RNeasy PowerSoil Total RNA Kit, QIAGEN). Genomic DNA was removed from total RNA (TURBO DNA-free Kit, Ambion, USA), and the remaining RNA was further concentrated (RNeasy MinElute Cleanup Kit, QIAGEN). The extracted RNA (with rRNA removed by Ribo-Zero rRNA Removal Kit, Illumina, USA) was subjected to metatranscriptomic sequencing in GENEWIZ Inc., Suzhou, China (details of library construction and sequencing in Supplementary Information Note 1).

### Phylogenetic analysis of MBG-D archaeal 16S rRNA gene sequences

A total of 3133 MBG-D archaeal 16S rRNA gene sequences (>1200 bps) were downloaded from SILVA SSURef 128 and parsed by a homemade Perl script to acquire their “isolation source” and “note” from corresponding gbk files [19]. The sequences were aligned by SINA [20], filtered by 50% sequence consensus and ssuref:archaea filters in ARB [21] (stored as “SSU_MBG-D.arb”), and dereplicated at the 97% level by QIIME [22]. The remaining sequences were used to construct phylogenetic trees with *Thermoplasma volcanium* GSS1 as an outgroup by RAxML-HPC v8 on XSEDE (CIPRES gateway) using “-T 4 -f a -c 25 -N 1000 -m GTRCAT -p 12345 -x 12345” and IQ-TREE 1.5.5 (Web server) using “-st DNA -m GTR+G4+F -bb 1000 -alrt 1000” [20, 23, 24]. The final tree was visualized by iTOL [25]. Clades with <0.36 branch length were assigned as subgroups and supporting bootstrap values were also taken into consideration. The environmental category, salinity, and acidity conditions were acquired from the sequence metadata. A total of 8503 MBG-D 16S rRNA gene sequences (>900 bp) were downloaded from the SILVA SSURef 128 and assigned to subgroups in “SSU_MBG-D.arb” by the ARB parsimony quick-add method after sequence alignment and column filtering (the same aligning and filtering method as that in building the backbone tree described above) [21]. Sequences originating from one study were regarded as one library, and environmental information parsed from NCBI was assigned to each library. The indicator lineages (ILs; MBG-D subgroups) for environments were calculated by IndVal in R package labdsv [26], which combines relative abundance and relative frequency to identify indicators significantly associated with environments (only studies with >5 sequences were included). The relative abundances of ILs were visualized by “polarHistogram.R” (https://github.com/chrislad).

### Meta-analysis and community networks of MBG-D archaea from sediments

Sediment archaeal 16S rRNA gene sequences were retrieved by “16S AND 600:2000 [Sequence Length] AND archaea [Organism] AND rrna [Feature key] AND isolation_source [All fields] NOT (genome OR chromosome OR plasmid)” in NCBI nucleotide database and aligned by SINA with SILVA taxonomy assigned (those without taxonomic assignments were excluded) [29,022 retrieved with 26,394 left after filtering (conducted on Nov 26, 2017)] [20]. Environmental conditions were assigned to individual studies according to sequence metadata in the same way as described above. QIIME scripts were used to make operational taxonomic unit (OTU) tables at 97% cutoff level with the default settings: one with each study having >30 sequences (“over30_OTU table” with 177 studies), one with each study having >10 MBG-D sequences (“MBG-D_over10_OTU table” with 58 studies) (script details in Supplementary Information Note 2) [22]. Species abundance distribution (SAD) and index of dispersion (IoD) plots were calculated to reflect the occurrence and abundance pattern and the dispersion pattern of archaeal lineages, respectively, based on the “over30_OTU table.” The beta-diversity patterns based on “MBG-D_over10_OTU table” were reflected in categories of salinity, environments, and seep condition, with 1000 permutations of the adonis test. Co-occurrence network analysis was performed according to the previous methods [27, 28] (details in Supplementary Information Note 3) based on “over30_OTU table.” The observed network reflected positive correlations (edges) among OTUs (nodes) with Spearman’s *ρ* > 0.4 and Benjamini–Hochberg adjusted *p* value <0.01. The observed network ended up with 205 nodes and 571 edges, and identically sized Erdös–Réyni (ER) random networks were simulated for 1000 times for comparison of network topological properties. The network construction, property characterization and visualization, and random network stimulation were conducted by R packages (vegan and igraph) and software Gephi [29–31].

### Metagenomic assembly, binning, and annotation

The 150 bp pair-end raw reads from Illumina HiSeq were dereplicated by a Perl script implemented in SeqTools (Genome Research Ltd.), then subjected to Sickle to trim low-quality reads with the default settings (https://github.com/najoshi/sickle). Clean reads for individual samples were applied in idba v1.1.1 for de novo scaffold assembling separately [32], with the settings of “--mink 65 --maxk 145 --step 10.” Obtained assemblies were deposited in the DOE-JGI IMG database and annotated by the DOE-JGI Microbial Genome Annotation Pipeline (MGAP v.4) [33]. In the first step, initial binning was conducted by MaxBin v2.2.1 with the default setting and the minimum contig length as 1000 bp [34]. Then CheckM v1.0.7 was used to assess the completeness and contamination of all MAGs and provide the placement of MAGs in the concatenated marker protein tree [35]. Based on the taxonomic information given by the default reference genomes and MAGs or single-cell amplified genomes (SAGs) within Thermoplasmata in the concatenated marker protein tree, MAGs affiliated with the Thermoplasmata clade were picked. Next, all potential Thermoplasmata MAGs from three samples, together with other reference MBG-D MAGs, SAGs, and fosmids affiliated with Thermoplasmata, were used as the reference for mapping raw reads by BBmap using “minid” as 0.6 (details in Supplementary Information Note 4) [36]. The properly paired reads mapping on the reference were dereplicated and trimmed and subsequently re-assembled by the same method described above. MaxBin v2.2.1 was applied to bin above sub-assemblies with the minimum contig length as 2000 bp. MAGs with >50% completeness were used and manually curated to reduce the contamination and strain heterogeneity. Finally, MAGs were translated by Prodigal v2.6.3 and annotated by non-redundant NCBI protein database (NCBI nr database, updated by Oct 4, 2016), BlastKOALA, and EggNOG v4.5.1 (HMMER mapping mode) with default settings [37–40]. The peptidases were recognized by the MEROPS database and also confirmed by the annotation of Pfam using InterProScan v5.21-60.0 and the top hit result using the nr database [40–42]. Peptidases with extracellular signal peptides were predicted using POSRTb and PRED-SIGNAL, and only congruent results from both of them led to assigning an extracellular peptidase [43, 44].

### Phylogenetic analysis of MBG-D genomes

The alignment of 43 concatenated phylogenomic markers, including all MBG-D MAGs and reference genomes (85 genomes in total), were processed by the CheckM software [35]. Only the MBG-D MAGs with completeness >70% were considered, and concatenated alignment sequences with <25% informative sites were excluded except for the reference genomes. Columns with >90% gaps along the alignment were deleted. The refined concatenated alignment was subjected to RAxML-HPC BlackBox on XSEDE (CIPRES gateway) for phylogenomic tree construction with a bacterial genome (*Acidimicrobium ferrooxidans* DSM 10331) as the outgroup and using “-m PROTCATLG -f a -N autoMRE” settings [23, 45].

The alignment of 16S rRNA genes was double filtered by 50% MBG-D sequence consensus and ssuref:archaea filters in ARB [21]. The phylogenetic tree of 16S rRNA genes was constructed by RAxML-HPC BlackBox on XSEDE (CIPRES gateway) using “-m GTRCAT -f a -N autoMRE” settings (details could be found in Supplementary Information Note 2).

### Metatranscriptomic analysis

Potential rRNA reads from raw data were filtered by SortMeRna v2.1b with the default settings [46]. Non-rRNA transcripts of individual samples were mapped to corresponding open-reading frames of MBG-D MAGs by Bowtie2 v2.2.8 with settings of “--no-mixed --no-discordant --no-dovetail --no-contain --no-overlap --very-sensitive.” The subsequent gene coverage of reads and Transcripts Per Kilobase Million (TPM) were calculated by “pileup.sh” (BBmap) [36] and “TPM-RPKM-calculator.py” (https://github.com/RichieJu520). TPM allowed comparisons of gene expression from sample to sample by normalizing different sequencing depths. The metatranscriptome from each sample was mapped to its corresponding MAGs, respectively.

## Results and Discussion

### Ecological significance of MBG-D archaea

**Fig. 1:**
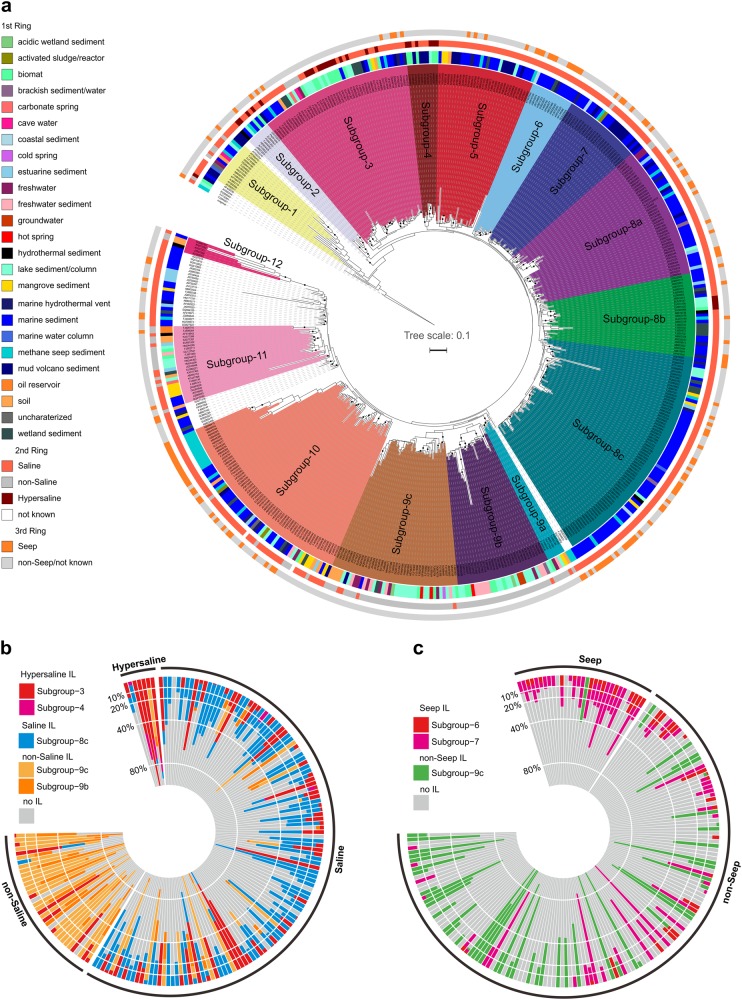
Phylogenetic tree of Thermoprofundales (**a**) and relative abundances of indicator lineages associated with environments (**b**, **c**). Dereplicated Thermoprofundales sequences (97% cutoff) from SILVA SSURef 128 was used to construct this RAxML-based phylogenetic tree. From the inside to the outside, the first ring denotes 25 environmental categories, the second ring denotes the salinity, and the third ring denotes the seep environment condition. Nodes with bootstrap values >75% were marked with black dots. The outgroup is the 16S rRNA gene sequence from *Thermoplasma volcanium* GSS1. The indicator lineages are inferred by their relative abundances and relative frequencies in all the libraries that they occur, based on statistical analysis. The significantly supported indicator lineages associated with salinity and seep conditions were used to plot the polar histogram figures depicting their abundance patterns in all the studied libraries

The phylogenetic tree of MBG-D archaea was reconstructed using 508 OTU representatives at 97% cutoff value, with 91.5% sequences assigned to 16 subgroups (Fig. 1a). MBG-D archaea have wide distribution with 16S rRNA gene sequences originated from 25 environmental categories. Among them, the top 3 most abundant environments were marine sediments, marine hydrothermal vents, and mangrove sediments, accounting for approximately 70% of the total sequences currently available in the database (Supplementary Table S1). The phylogenetic trees of subgroups are roughly congruent in the RAxML and IQ trees (Supplementary Information Note 2), indicating that subgroup topology remains largely stable, even with partial branch nodes with low bootstrap support. The IndVal function identifies the ILs (MBG-D subgroups) not only significantly associate with particular environments (*p* value < 0.05) but also constitute a large fraction of the lineages in their respective environments (Fig. 1b, c). The significant segregation of subgroups toward saline and seep condition echoes the previous research on Bathyarchaeota, in which the distinct evolutionary Bathyarchaeota subgroups have been found in freshwater and marine sediments, suggesting a niche-specific adaptation [47].

### Meta-analysis of sedimentary MBG-D archaea

**Fig. 2:**
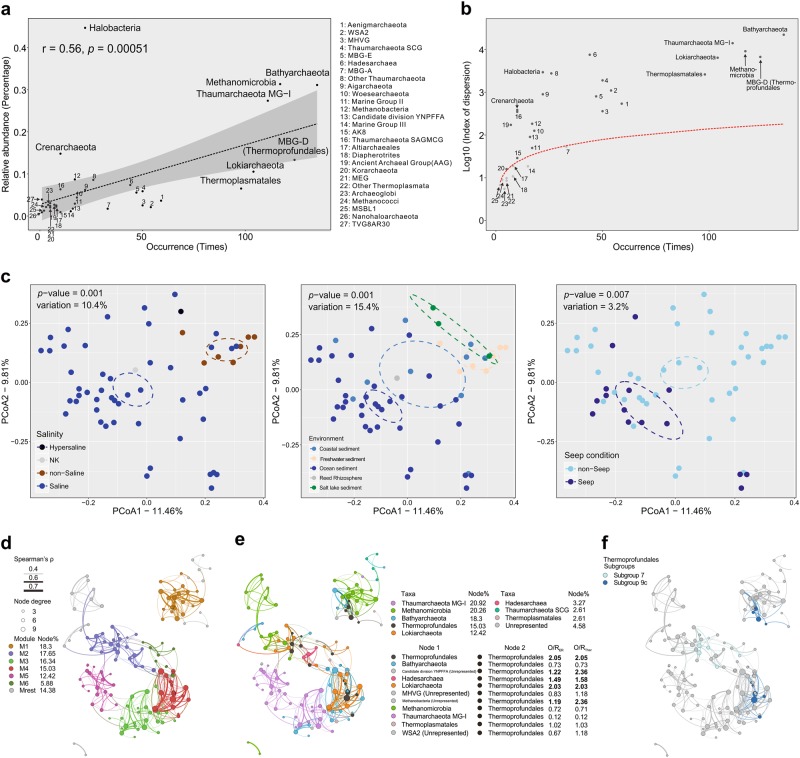
**a** Species abundance distribution (SAD) figure with relative abundances of archaeal lineages plotted against their occurrences in 177 studies (“over30_OTU table”). The vertical axis stands for the average relative abundance of one archaeal lineage across all libraries that they appear; the horizontal axis stands for the number of times this archaeal lineage being detected across all libraries. **b** Index of dispersion (IoD) figure with log_10_-transformed indices of dispersion of archaeal lineages plotted against their occurrences in 177 studies (“over30_OTU table”). Taxa with singletons are excluded from the IoD figure. The red line depicts the 0.5% confidence limit of chi-square distribution. Lineages below this line follow a Poisson distribution and are randomly distributed in the environment. **c** Beta diversity plots of 58 studies based on the “MBG-D_over10_OTU table.” Beta diversity was calculated by the unweighted Unifrac matrix method with a 1000-permutation adonis test. Subplots color-coded by salinity, environment, and seep condition are shown, with dashed-line circles representing 95% confidence intervals for groupings. Co-occurrence networks depict correlations among nodes that are affiliated within different modules (**d**) and different archaeal lineages (**e**). Nodes affiliated to Thermoprofundales subgroups are highlighted in the network (**f**). The ratio of observed co-occurring incidence between two archaeal lineages (*O*) over random co-occurring incidence (*R*) of that pair (*R*_ER_ is the mean value of the observed co-occurring incidences for 1000 identically sized Erdös–Réyni random networks; *R*_Theo_ is the theoretical co-occurring incidence calculated by giving the identical frequencies of archaeal lineages and random association between nodes) is an estimation of non-random association of two archaeal lineages; that is O/R ratio more than 1 stands for two archaeal lineages non-randomly associated (potentially reflecting a synergistic relationship) in the environment. Significant non-random associations of Thermoprofundales and other lineages are highlighted (with two *O*/*R* ratios significantly >1)

An updated collection of 23,194 sedimentary archaeal sequences from 177 studies (each study contains at least 30 sequences) was assigned to 36 archaeal lineages for SAD analysis, plotting relative abundance against occurrence (frequency of archaeal lineage in all studies) (Fig. 2a, Supplementary Table S2). A linear regression with significant support indicates that archaeal lineages of widespread distribution across studies have higher abundance (frequency of occurrence in sequence collection) than those of limited distribution in environments, similar to the previous report [47]. The archaeal lineages could be divided into two groups of over or under 75 occurrences, with one group as cosmopolitan lineages of persistent/abundant distribution, and another group as narrow lineages of rare/less abundant distribution (Fig. 2a). This phenomenon is consistent with the macroecological concept “jack-of-all-trades is master of all”, stating that cosmopolitan lineages could tolerate a wide range of environments and utilize a wide range of resources or commonly shared resources to become locally abundant in all environments [47–50]. The MBG-D archaea group is the second most frequent archaeal lineage, with 124/177 occurrences and 13.3% of relative abundance of 16S rRNA gene sequences in these studies, which outnumbers Thermoplasmatales (98/177, 6.5%) and other Thermoplasmata (5/177, 3.1%), suggesting that MBG-D archaea are a ubiquitous sedimentary archaeal lineage with an important ecological significance. The dispersion indices of archaeal lineages were plotted to test whether they follow a stochastic distribution (Poisson model) by comparing to a 0.5% confidence limit of chi-square distribution (Fig. 2b). The nine satellite lineages fell below the confidence limit, while the rest (the core lineages) were above it (*p* < 0.01), indicating a non-stochastic distribution among sedimentary environments. The MBG-D archaea, like the Bathyarchaeota investigated previously, appear to be core generalists non-randomly distributed across global sedimentary environments [47].

The MBG-D archaea from “MBG-D_over10_OTU table” were sorted into principal coordinates analysis (PCoA) ordination to look for associations between specific subgroups and particular environmental conditions (Fig. 2c). The salinity and environment category have large explanatory effects on the PCoA ordination with significant supports, and seep condition also shows significant influence on the PCoA ordination. These results indicate that salinity, habitat (also related to salinity), and seep condition have large influences on MBG-D archaea distribution. As evident in the ILs (the subgroups significantly associated with specific environments with statistical support) (Fig. 1), this probably results from the adaptation of particular subgroups to their corresponding eco-niches. Nevertheless, the potential physiological backgrounds of different subgroups, which might cause the different adaptation patterns, remain elusive. More studies on genomic and physiological profiles of MBG-D subgroups are encouraged to address their adaptive strategies toward different environmental conditions.

### Co-occurrence network of sedimentary MBG-D archaea

The co-occurrence network depicts potential close-interacting or niche-sharing relationships in which MBG-D archaea could be involved, by showing co-occurring patterns between OTUs with strong and significant correlations (Fig. 2d–f). The *C*-score test has indicated that the observed network (205 nodes and 571 edges) rejects the null model hypothesis of random co-occurrence, indicating that the observed network has fewer co-occurrences than expected by chance (containing segregated nodes in selective modules) (Supplementary Information Note 3). By comparing to 1000 times simulated ER random networks, the observed network has “small-world” properties, which means that, in this observed “small-world” network, nodes are more connected than in an identically sized random network [51]. Meanwhile, an MD (modularity degree) value > 0.4 suggests that the observed network has a modular structure [52]. The modules are suggested as segregated functional/ecological niches [53].

The MBG-D archaea have the fourth most abundant nodes (15.03%) in the observed network, occurring in 4 out of the 6 major modules. The exceptions are modules M3 and M5, which mainly represent assemblages of Thaumarchaeota and Bathyarchaeota (Fig. 2d, e). The co-occurrence incidences suggest that Lokiarchaeota have the highest non-random (*O*/*R*_ER_ and *O*/*R*_Ther_) association with MBG-D archaea. Meanwhile, Hadesarchaea also show significant non-random association with MBG-D archaea in the network (Fig. 2e). This relationship could probably result from a niche overlap, rather than a synergistic/syntrophic relationship. However, as with Lokiarchaeota, these associations are present in all modules containing these two lineages (Fig. 2d, e), and two MBG-D archaea subgroups from non-saline and saline origins (Fig. 2f) are also associated with Lokiarchaeota, which might support a potential synergistic/syntrophic relationship between MBG-D and Lokiarchaeota.

### Genomic properties, definition, and description of Thermoprofundales (MBG-D)

The obtained metagenomic sequencing data include three libraries of sizes 91.0, 88.6, and 86.0 gigabases for MaiPo-8, MaiPo-9, and MaiPo-11, respectively. Four MBG-D archaeal MAGs (M11B2D, 5.0× coverage; M8B2D, 12.0× coverage; M9B1D, 48.0× coverage; and M9B2D, 23.5× coverage) with genome completeness >75% were retrieved by metagenomic binning (Table 1). All the MBG-D MAGs resolved from Mai Po wetland and IMG deposited metagenomes had high genome completeness and low contamination and strain heterogeneity, compared to former MAGs and SAGs [5, 14].

**Table 1:** Overview of genomic statistics of Thermoprofundales MAGs

SAG/MAG	M8B2D	M9B1D	M9B2D	M11B2D	3300003432D	SG8-52-3	SG8-52-4	SM1-50	SCGC AB-539-N05	SCGC AB-539-C06	SCGC AB-540-F20
Copies of individual markers											
0	14	33	29	48	29	23	34	11	74	130	103
1	122	154	152	130	154	162	143	173	108	53	46
2	13	1	6	10	4	3	9	4	6	5	0
3	0	0	1	0	1	0	2	0	0	0	0
4	0	0	0	0	0	0	0	0	0	0	0
5+	0	0	0	0	0	0	0	0	0	0	0
Completeness (%)	88.79	81.55	89.34	77.78	79.6	85.2	77.2	94.74	51.42	21.46	35.51
Contamination (%)	7.32	0.8	5.6	7.8	4.8	2.4	7.6	2.4	1.81	1.64	0
Strain heterogeneity (%)	0	0	0	0	0	0	6.67	0	83.33	20	0
Genome size (bp)	2,555,641	1,692,085	2,781,214	1,712,033	2,023,941	1,909,404	2,246,271	2,091,705	801,028	593,453	1,037,251
Estimated genome size (bp)	2,878,298	2,074,905	3,113,067	2,201,122	2,542,639	2,241,085	2,909,677	2,207,837	1,557,814	2,765,391	2,921,011
N50 (bp)	7232	6825	14,196	5506	29,868	30,406	31,397	33,816	27,065	10,367	12,904
Longest scaffold (bp)	35,826	55,446	64,248	21,103	148,374	131,306	104,598	111,154	73332	48190	77,009
Scaffold number	458	298	350	356	135	84	91	94	99	104	172
Mean scaffold length (bp)	5580.00	5678.14	7946.33	4809.08	14,992.16	22,731.00	24,684.30	22,252.18	8091.19	5706.28	6030.53
GC (%)	32.2	31.4	42.3	43.6	43.2	30.4	31.6	39.0	36.5	35.0	35.6
GC standard deviation (%)	3.1	2.4	2.5	2.5	1.9	1.8	2.3	2.8	2.7	2.9	2.9
Coding density (%)	86.1	88.8	88.9	90.4	88.3	91.0	90.7	85.3	90.7	83.3	85.7

The first four MAGs were reconstructed from metagenomic DNA from Mai Po wetland sediments. 3300003432D was binned from the metagenomic sequencing deposit (IMG: 3300003432, surface sediment sample from Twitchell Island in the Sacramento Delta). SG8-52-3, SG8-52-4, and SM1-50 were originated from ref. [14], binned from sulfate-reducing zone (the former two MAGs) and sulfate-methane transition zone (the last MAG) of sediment profile from White Oak River estuary, North Carolina. The last three SAGs were originated from ref. [5], and the cells are sorted from 10-cm depth organic-rich marine sediments, Aarhus Bay, Denmark. The genomic property data of SAGs and MAGs were calculated by CheckM. Because of the low completeness of the three SAGs, only eight MAGs are included in the downstream genomic analysis

**Fig. 3:**
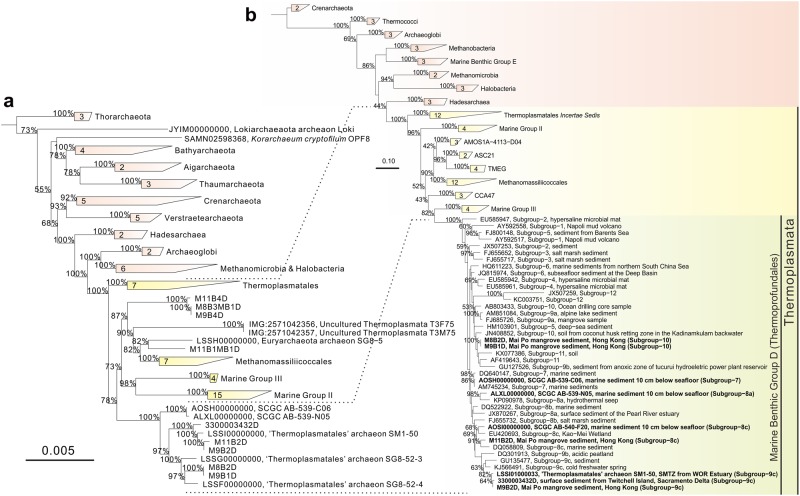
Phylogenetic tree placing Thermoprofundales archaea into the Thermoplasmata. **a** Phylogenetic tree based on 43 concatenated markers. This concatenated protein alignment for phylogenomic reconstruction was obtained from the intermediate files in the process of placing MAGs and reference genomes into the reference tree by CheckM. **b** Phylogenetic tree based on 16S rRNA gene sequences of representative Thermoprofundales archaeon sequences from each subgroup and the reference archaeal groups. SAG- or MAG-derived sequences are highlighted, and the dashed lines stand for the corresponding relationship between two trees. The method for tree reconstruction is detailed in Supplementary Information Note 2. The old names of “Thermoplasmatales” archaeon in both trees are used, but they are in fact affiliated to Thermoprofundales as figured out in this study

Phylogenetic analyses of both the 43 concatenated markers and 16S rRNA genes confirmed the placement of MBG-D archaea into Class Thermoplasmata, within Phylum Euryarchaeota (Fig. 3). Furthermore, the sequence similarities among all available 16S rRNA genes of MBG-D archaea indicated that this archaeal group should be proposed as an order rather than a class (Izemarchaea) according to sequence identity range (Table 2) [19, 54, 55]. Based on the above genome properties and phylogeny of MBG-D archaea, we hereby proposed this uncultured archaeal group as a new archaeal order, Thermoprofundales (Ther.mo.pro.fund.a’les. N.L. suff. -*ales* ending designing an order name; N.L. masc. pl. n. *Thermoprofundales*, the order denoting MBG-D archaea, affiliated to Thermoplasmata). This nomenclature of Thermoprofundales is derived from the class name of Thermoplasmata, which is represented by the thermophilic acidophilic strains of *Thermoplasma* and *Picrophilus* [56]. Since Thermoprofundales was first discovered in marine benthic sediments with various widespread distribution and enigmatic ecological roles, L. adj. profundus could generally describe its living preference [9].

**Table 2:** Proposed taxonomic level based on sequence identity range

Taxonomic group	Alternative name	Median sequence identity^a^	Median sequence identity^b^	Proposed taxonomic level based on sequence identity range^c^
Bathyarchaeota	Miscellaneous Crenarchaeotal Group (MCG)	82.6	78.2	Phylum
Lokiarchaeota	Marine Benthic Group B (MBG-B)	87.1	81.2	Phylum-Class
MSBL1	Persephonarchaea (proposed)	89.2	86.9	Class-Order
WSA2	*Candidatus* Methanofastidiosa	75.6	71.5	Phylum
Hadesarchaea	South African Gold Mine Euryarchaeotic Group (SAGMEG)	86.7	82.8	Class
Thermoprofundales	Marine Benthic Group D (MBG-D)	91.8	88.1	Order-Family
Marine Group II	Thalassoarchaea (proposed)	80.0	77.3	Phylum
Marine Group III	Pontarchaea (proposed)	90.0	78.4	Class-Order

^a^Median sequence identity of representative sequences with 0.97 similarity cutoff from SILVA database with sequence length >1400 bp and pintail value >75 (Aug 10, 2017 updated). The sequence identity matrices for individual taxonomic group were calculated by BioEdit with default settings [87]

^b^Median sequence identity of representative sequences with 0.97 similarity cutoff from SILVA database with sequence length >1200 bp and pintail value >75 (Aug 10, 2017 updated)

^c^The sequence identity ranges for different taxonomic levels are according to ref. [55]

### Wood–Ljungdahl (WL) pathway with two types of C_1_ carrier

**Fig. 4:**
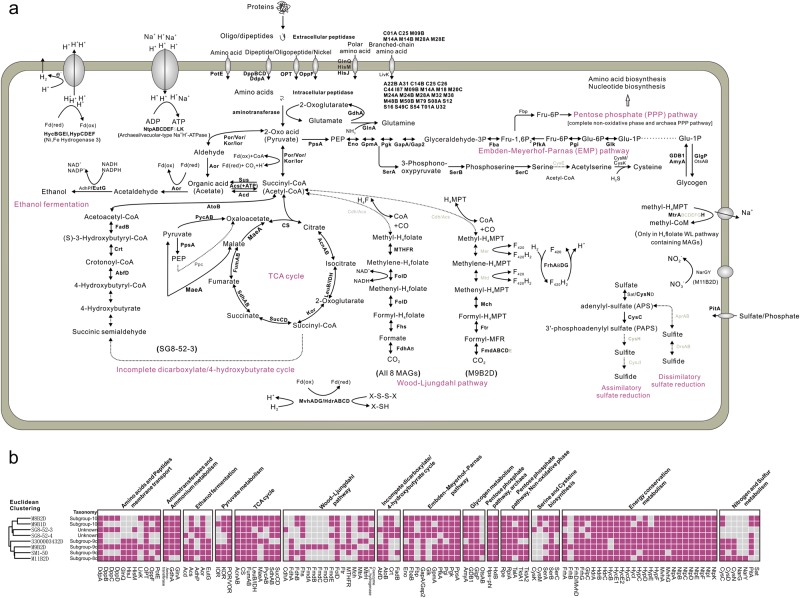
Reconstructed key metabolic pathways of Thermoprofundales based on eight MAGs (**a**). Enzymes identified in over half of the eight MAGs are labeled in bold, those identified in less than half of the eight MAGs are labeled normally, and those for which no genes were found in any MAGs are labeled in gray. The extracellular and intracellular peptidases listed are those occurring in more than six out of the eight MAGs. Pathways for which no genes were found are drawn with dashed lines. The H_4_MPT C_1_ carrier-dependent WL pathway and incomplete dicarboxylate/4-hydroxybutyrate cycle are depicted according to MAG M9B2D and SG8-52-3, respectively. Euclidean-clustering-based gene presence heatmap (**b**). The presence/absence of protein encoding genes are represented by the heatmap, and the Euclidean clustering among all the MAGs was based on the presence–absence binary matrix of the metabolic genes

Thermoprofundales MAGs in this study mainly come from subgroup 8c, 9c, and 10 (Figs. 3 and 4), which cover both saline and non-saline ILs. As genome representatives, the MAGs enable us to provide comprehensive insights on the metabolic properties of Thermoprofundales. The presence/absence matrix of metabolic genes showed two general clusters (Fig. 4); however, the LEfSe (linear discriminant analysis effect size) analysis indicated that no significant gene repertoire distribution associated with different subgroups was detected (Supplementary Information Note 5). Therefore, we integrated all the MAGs in the following genomic content analysis.

Among the eight available MAGs, M9B2D was the only one containing genes for the tetrahydromethanopterin (H_4_MPT)-WL pathway. This MAG contained putative genes for subunits of the formyl-methanofuran dehydrogenase (Fmd) in the carbonyl-branch of the WL pathway and the formyl transferase (Frt) and 5,10-methenyl-H_4_MPT cyclohydrolase (Mch) of the methyl-branch but lacked the rest protein-coding genes (Fig. 4). On the other hand, all the eight Thermoprofundales MAGs (including M9B2D) contained putative genes for intermediate enzymes in methyl-branch of H_4_folate-WL pathway, similar to typical bacterial acetogens [57]. No genes for CO-dehydrogenase/acetyl-CoA synthase (Cdh/Acs) were identified in all MAGs, except for a putative CdhA gene (converting CO_2_ to CO) in SG8-52-3. Cdh/Acs is important for the carbonyl branch of WL pathway for reducing CO_2_ to CO and combining CO with a methyl residue to produce acetyl-CoA [58, 59]. Notably, genes encoding Cdh/Acs complex within the WL pathway are commonly absent in many available genomes affiliated to Thermoplasmatales, MG-II, and MG-III (Supplementary Information Note 5), probably because their Cdh/Acs complexes share less sequence similarity with those from other lineages of Euryarchaeota and TACK superphylum.

### Incomplete dicarboxylate/4-hydroxybutyrate cycle

Several Thermoprofundales MAGs encoded enzymes involved in the hydroxybutyrate (HB) part of the dicarboxylate/4-hydroxybutyrate cycle (abbreviated as DC/4-HB or dicarboxylate/hydroxybutyrate cycle), which converts one succinyl-CoA molecule through 4-HB to two acetyl-CoA molecules. Among the MAGs, SG8-52-3 had the most complete inferred pathway for the HB part (Fig. 4). However, it still lacked succinyl-CoA reductase, succinic semialdehyde reductase (NADPH), and 4-hydroxybutyrate-CoA ligase, which would catalyze the steps from succinyl-CoA to 4-hydroxybutyryl-CoA. SG8-52-3 does contain a candidate 4-hydroxybutyryl-CoA dehydratase gene, the maker gene for catalyzing the radical-mediated dehydration from 4-hydroxybutyrate-CoA [60, 61]. Similar genomic evidence could be found in *Archaeoglobus fulgidus* DSM 4304, *A. fulgidus* DSM 8774 [60, 62] and *Ignicoccus hospitalis* KIN4/I [63], where their genomes contain nearly all genes in the DC/4-HB cycle but all lack potential genes of succinyl-CoA reductase and succinic semialdehyde reductase (NADPH), similar to Thermoprofundales. Thus evidence suggests that Thermoprofundales might have the effective DC/4-HB cycle and the apparently missing genes may be too divergent from the known ones to be detected by the methods used. Nevertheless, enzymatic analyses and substrate incorporation experiments are required to further confirm the function of the DC/4-HB cycle.

Interestingly, SG8-52-3 is predicted to encode an alternative decarboxylation pathway [60, 63]. It apparently lacks a phosphoenolpyruvate carboxylase, which produces oxaloacetate and fixes one molecule of CO_2_. Alternatively, it could use one of the following bypasses: (i) converting pyruvate to malate via the catalysis of (S)-Malate:NAD(P)^+^ oxidoreductase, and then using the subsequent tricarboxylic acid (TCA) cycle to generate succinyl-CoA; (ii) directly carboxylating pyruvate to oxaloacetate via the catalysis of pyruvate carboxylase, and then applying the normal TCA cycle to generate succinyl-CoA. Genes potentially encoding both the DC/4-HB cycle and the TCA cycle were found in nearly all the Thermoprofundales MAGs and were especially complete in SG8-52-3, which is similar to the scenario in the Thermoproteales genome [64]. They could both be used for autotrophic CO_2_ fixation; however, when both exist in one genome, the DC/4-HB cycle is expected to operate actively rather than the reductive TCA cycle in Thermoproteales and Desulfurococcales [64]. In our case, the TCA cycle may operate only in the oxidative direction for heterotrophic acetyl-CoA utilization, because that the reductive TCA cycle markers *aclAB* and *frdAB* were not found in any of the MAGs [65]. Combined with the above analysis, the WL pathway and DC/4-HB cycle within Thermoprofundales probably both participate in the autotrophic direction.

### Extracellular and intracellular peptidases, amino acid, and carbohydrate metabolism

Consistent with earlier studies, putative genes for clostripain (C11), gingipain (C25), interpain (C10), and legumain (C13) were each found in at least one of these MAGs [5, 14]. Possible genes for other extracellular peptidases, including collagenase H (M09B), carboxypeptidase A1&E (M14A & B), and aminopeptidase S&Ap1 (M28A & E) were each found in at least six of eight MAGs (Supplementary Table S3). Amino acid/polar amino acid and oligopeptide transporters and ABC-type dipeptide/oligopeptide/nickel transport systems were discovered in at least half of eight MAGs (Supplementary Table S4). A candidate LivK, the substrate binding protein of the branched-chain amino acid transport systems, could only be identified in M8B2D and M9B2D (Supplementary Table S4). There were 26 types of intracellular peptidases discovered in the at least 6 of the 8 MAGs (Supplementary Table S3). A variety of aminotransferases were present for transferring amino-groups from amino acids to 2-oxoglutarate and generating glutamate (Supplementary Table S4). Pyruvate/ketoisovalerate:ferredoxin oxidoreductase (Por/Vor) and 2-oxoglutarate/2-oxoacid:ferredoxin oxidoreductase (Kor) were present in six to eight MAGs, and indolepyruvate:ferredoxin oxidoreductase (Ior) was present in four out of the eight MAGs. All of them may be responsible for breaking down amino acids [5, 66] and mediate the complete pathways of transporting and breaking down proteins to acetyl-CoA, generating intermediate CO_2_, and transferring electrons to oxidized ferredoxins for energy recycling (Fig. 4).

Interestingly, none of the Thermoprofundales MAGs contained identifiable genes for ABC-type sugar transport system proteins or featured proteins of carbohydrate assimilation, highlighting that using proteins rather than carbohydrates for carbon and energy sources may be a common metabolic property of Thermoprofundales [14]. On the one hand, acetate from extracellular import and intermediates in the protein degradation pathway could be converted into acetyl-CoA by AMP-forming acetyl-CoA synthetase (EC: 6.2.1.1). On the other hand, acetate could be produced by ATP-producing acetyl-CoA synthetase (EC: 6.2.1.13) and acetaldehyde could be produced from acetate at the cost of reduced ferredoxins by aldehyde:ferredoxin oxidoreductase (Aor) (Fig. 4). Six out of the eight MAGs contained putative genes for iron-containing alcohol dehydrogenase family proteins EutG or AdhP, and SM1-50 has both of them (Fig. 4). The EutG and AdhP candidates are both of very low (<50%) sequence identity with their homologs in the nr database and form a branch phylogenetically distinct from other bacterial clades in the phylogenetic tree (Supplementary Information Note 5). Both the conserved domain and functional site analyses suggest that they acquire the ethanol-producing functions (Supplementary Information Note 5). Over half of the eight Thermoprofundales MAGs contained a complete Embden–Meyerhof–Parnas (EMP) pathway for both gluconeogenesis and glycolysis. They encoded nearly all the key genes for the non-oxidative phase of pentose phosphate pathway, in which ribose-5P, the important intermediate for both DNA and RNA biosynthesis, and erythrose-4-P, the precursor of aromatic amino acids, were produced. The Thermoprofundales MAGs also encoded serine and cysteine biosynthesis pathways, branching off from the gluconeogenesis direction of the EMP pathway. This suggests that Thermoprofundales maintain a well-established nucleic acid and amino acid anabolic metabolism for essential cell life activities [67].

### Nitrogen and sulfur metabolisms

The majority of the eight MAGs contained phosphate/sulfate permeases for transporting phosphate/sulfate into cells (Fig. 4). Putative genes for the first two steps of assimilatory sulfate reduction, reducing sulfate to sulfite via adenylyl-sulfate and 3′-phosphoadenylyl sulfate, could be found in the majority of the eight MAGs. However, the two steps of reducing sulfite to sulfide via the catalysis of CysH and CysJI are missing. This indicates that Thermoprofundales could probably depend on sulfate assimilation rather than only depending on incorporating sulfur sources from sulfur-containing peptides [14, 68], a strategy to assimilate more sulfur for biosynthesis from sulfate flux derived from upper oxic layers. M11B2D, resolved from intertidal mudflat sediment, contained putative nitrate reductase (NarGY) genes, indicating that Thermoprofundales from this eco-niche could participate in the initial step of denitrification or dissimilatory nitrate reduction to ammonia. The closest NarGY protein hits in the nr database were of *Sulfuricurvum* (bacterial) origin, instead of archaeal origin. Nevertheless, the *nar* gene was inferred to emerge before the divergence of bacteria and archaea during the pre-oxic times, and horizontal gene transfer between archaea and bacteria could also blur the phylogenetic relationship between *nar* and 16S rRNA genes [69].

### Transcriptomic pattern

After processing read quality control (read dereplication and low-quality read trimming as described above) and deleting rRNA reads, three metatranscriptomic libraries were obtained from three sediment samples, which are of sizes 8.4, 8.7, and 12.0 gigabases for MaiPo-8, MaiPo-9, and MaiPo-11, respectively. The transcripts of MAGs from this study reflect the expression level of certain pathways and genes (Table 3, Supplementary Table S5). Key genes of acetate and amino acid utilization and peptide transportation were effectively expressed, which corroborates the metabolic properties deduced from metagenomic analysis. Genes assigned to proteins involved in the WL pathway, EMP pathway, and energy conservation were also highly expressed, which indicates that pathways for cell function and genes for building cell structures were also active in Thermoprofundales. The extracellular and intracellular peptidases, including C01A, C14B, C25, M09B, and etc., were expressed in these MAGs. Specifically, M09B (collagenase) as one of the most abundant extracellular peptidases in Thermoprofundales genomes were also mostly expressed in all four mangrove MAGs (TPM ranging from 3793.3 to 50,740.9). Collagen is the most abundant and ubiquitous material making up the extracellular matrices of animals, and nearly 30% of the total protein of animals are made of collagens [70]. Many collagenolytic-protease-secreting bacteria have been isolated from terrestrial and marine sediments [70]. It is suggested that collagen degradation by extracellular collagenolytic proteases from various environmental bacteria is an important biological process for the release of fixed nitrogen (such as that within animal carcasses) into the global nitrogen cycle [70]. The high expression of collagenase of Thermoprofundales suggests their role of utilizing detrital proteins in global sediments.

**Table 3:** Transcriptomic level of proteins in MAGs from this study

Protein ID	TPM	nr annotation	Pathway	Function
M8B2D_scaffold_645_2	422,998.6	Glyceraldehyde-3-phosphate dehydrogenase	Embden–Meyerhof–Parnas pathway	Catalyze gluconeogenesis/glycolysis
M8B2D_scaffold_734_2	13,212.5	Acetyl-coenzyme A synthetase	Ethanol fermentation	Utilize acetate
M8B2D_scaffold_66_13	9707.6	(HdrA) NADPH-dependent glutamate synthase beta chain-like oxidoreductase	Energy conservation metabolism	Energy conservation
M8B2D_scaffold_670_8	3091.0	(HdrA) Pyridine nucleotide-disulfide oxidoreductase, partial	Energy conservation metabolism	Energy conservation
M9B2D_scaffold_1366_5	80,096.5	(DdpA) family 5 extracellular solute-binding protein	Amino acids and Peptides membrane transport	Transport peptide/nickel into cell
M9B2D_scaffold_3574_1	12,959.8	2-Oxoglutarate synthase subunit alpha	Pyruvate metabolism	Break down pyruvate in degradation of amino acids
M9B2D_scaffold_268_3	4935.3	Hydrogenase	Energy conservation metabolism	Energy conservation
M9B2D_scaffold_547_2	2852.8	Formylmethanofuran dehydrogenase subunit A	Wood–Ljungdahl pathway	fixing CO_2_
M11B2D_scaffold_242_4	36,092.9	NADH dehydrogenase	Energy conservation metabolism	Energy conservation
M11B2D_scaffold_698_2	11,166.4	Acetyl-coenzyme A synthetase	Ethanol fermentation	Utilize acetate

	TPM	nr annotation	MERPOS family	Location
M8B2D_scaffold_243_7	24,364.3	—	C25	Extracellular
M8B2D_scaffold_328_11	16,526.7	PKD domain-containing protein	M09B	Intercellular
M8B2D_scaffold_290_3	13,560.8	Hypothetical protein AYK22_04980	C01A	Extracellular
M8B2D_scaffold_77_9	13,397.4	—	C25	Extracellular
M8B2D_scaffold_308_3	7856.4	—	C25	Extracellular
M8B2D_scaffold_432_4	3793.3	PKD domain protein	M09B	Extracellular
M9B1D_scaffold_105_29	50,740.9	PKD domain-containing protein	M09B	Extracellular
M9B1D_scaffold_577_3	42,987.6	—	C25	Intercellular
M9B1D_scaffold_412_9	14,720.0	—	C25	Extracellular
M9B2D_scaffold_561_4	24,221.1	PDK repeat-containing protein	M09B	Extracellular
M9B2D_scaffold_235_10	14,055.0	Aminopeptidase	M29	Intercellular
M9B2D_scaffold_1736_5	11,657.8	6-Aminohexanoate hydrolase	S12	Intercellular
M9B2D_scaffold_156_3	8305.5	Protein containing Por secretion system C-terminal sorting domain	C25	Extracellular
M9B2D_scaffold_1754_3	5715.4	—	M23B	Intercellular
M9B2D_scaffold_51_2	4215.9	—	C25	Extracellular
M9B2D_scaffold_83_12	4017.3	PDK repeat-containing protein	M09B	Extracellular
M9B2D_scaffold_161_3	2558.7	—	C25	Extracellular
M11B2D_scaffold_542_7	50,210.9	PKD domain protein	C25	Intercellular
M11B2D_scaffold_792_1	23,848.1	—	C25	Intercellular
M11B2D_scaffold_2177_1	12,159.8	S8 family peptidase	S08A	Intercellular
M11B2D_scaffold_496_3	11,771.9	Peptidase C1A, papain C-terminal	C01A	Extracellular
M11B2D_scaffold_512_1	7988.1	F5/8-type C domain-containing protein	M09B	Extracellular

Detailed table refers to Supplementary Table S5. Protein abbreviations within parentheses are according to arCOG annotations of EggNOG

### Biogeochemical roles and metabolic summaries

Genomic studies have suggested that Thermoprofundales could effectively use proteins for biosynthesis and cell activity but lack pathways for transporting and assimilating carbohydrates [14]. Since mangrove leaves and wood debris are mainly made of lignocellulose, their colonization and degradation by heterotrophic bacteria and fungi should release plant-derived oligosaccharides to mangrove sediments [71–73]. Although oligosaccharides might not be directly utilized by Thermoprofundales, they could be utilized by other heterotrophs to generate intermediates or products that would be subsequently utilized by Thermoprofundales. According to the above genomic studies and previous results [18], Thermoprofundales in the Mai Po wetland, which occupy 32% relative abundance among archaeal communities, could fuel the turnover of organic matter, especially for detrital proteins, together with other microbial heterotrophs to recycle and conserve nutrients and maintain microbe–nutrient–plant relationship and extensive food web of the ecosystem [71].

Given their predicted potential for protein remineralization and for CO_2_ fixation by the WL pathway or the dicarboxylate/4-hydroxybutyrate cycle, it is reasonable to suggest that the global distribution of Thermoprofundales may be due to their mixotrophic lifestyle (Figs. 2 and 4) [5, 14]. Beyond that, they also appear to have acquired the ability to assimilate sulfur from sulfate or protein-derived sulfur compounds. This metabolic capacity may have enabled Thermoprofundales to effectively adapt to benthic sediment environments with various carbon substrate conditions, especially in the energy-limited deep subsurface [5, 14]. Thermoprofundales could produce acetate and ethanol, subsequently providing small molecular substrates for heterotrophic microorganisms and acetoclastic methanogens. Acetogenesis is the energetically more favorable metabolic pathway for organic substrate utilization, so that Thermoprofundales may serve as effective organic matter transformers in benthic sediment environments [74, 75].

Genomic reconstruction has suggested that Lokiarchaeota might be not strictly autotrophic but could derive energy from acetogenesis on hydrogen, formate, or other organics [76, 77]. Our results raise the possibility that Thermoprofundales and Lokiarchaeota might use similar substrates in both marine and terrestrial sedimentary environments, synergistically work for carbon remineralization from sediments, and produce more labile compounds for other microorganisms. Nevertheless, co-occurrence networks do not always effectively predict actual classical ecological networks, in which the interactions are represented by direct observations or experiment manipulations [78], thus the omics-based profiling and culture-dependent approaches are needed to further test and understand the potential synergistic/syntrophic relationship. Furthermore, Woesearchaeota AR20, Diapherotrites AR10 (DPANN superphylum), Thorarchaeota, and Lokiarchaeota all contain alcohol dehydrogenase for ethanol synthesis from acetyl-CoA and could produce acetate and ethanol as fermentation products [79, 80]. Thermoprofundales, together with other major archaeal groups, such as Bathyarchaeota [5, 15, 74, 81], Thorarchaeota [82, 83], Lokiarchaeota [79, 84], Woesearchaeota [80, 82], Diapherotrites [80, 85], Marine Group II/III [86], and etc., have a wide spectrum of organic-matter-utilizing capacity and synergistically fuel the carbon turnover in natural environments, providing new microbial biogeochemical insights on the carbon and nutrient flow in global scale.

## Supplementary Information

Supplementary Information

Table S1 _Dataset 1

Table S2 _Dataset 2

Table S3 _Dataset 3

Table S4 _Dataset 4

Table S5 _Dataset 5
