# Supplementary material for: Genomic and transcriptomic insights into the ecology and metabolism of benthic archaeal cosmopolitan, Thermoprofundales (MBG-D archaea)
Source: ISME J. 2018 Dec 4;13(4):885–901. doi: 10.1038/s41396-018-0321-8 (PMC6461988; doi:10.1038/s41396-018-0321-8)
Supplement: Supplementary file 1 — Supplementary Information [file 41396_2018_321_MOESM1_ESM.docx]

**Supplementary Information**

**1. Supplementary Note 1: Sample sites description, physicochemical properties, and processing details**

Sediments were acquired on Sep 12, 2014 from the Mai Po Nature Reserve after the raining season of Hong Kong. Mai Po Nature Reserve was located in the northwestern part of Yuen Long Plain, the largest alluvial plain in northwestern part of Hong Kong. It is facing the Deep Bay, which connects the Shenzhen River estuary to the Pearl River estuary, and subjected to salt seawater influence. In the margin of Mai Po wetland, the mixture of freshwater and salt water provides valuable habits for a subtropical mangrove forest, and beyond the outline of mangrove forest, it is characterized as an area of intertidal mudflats, which is strongly influenced by the daily tide. The weather of the sampling date was strongly represented as the rainy season of Hong Kong. The average values of mean temperature and total daily rainfall before the sampling date were 29°C and 16.6 mm. MaiPo-8 sediment sample was collected from mangrove forest at the depth of 10-15 cm, MaiPo-9 sediment sample was collected from the same site of MaiPo-8 at the depth of 20-25 cm and MaiPo-11 sediment sample was collected from intertidal mudflats at the depth of 13-16 cm. After sampling, the bulk sediments were collected and sealed into plastic bags immediately, and stored in pre-cold sampling box temporarily; shipped to lab in a short time. For each sample, 5 g fresh sediments were subjected to further physicochemical parameter measurements and the rests were stored for long time storage in -20°C refrigerator for DNA isolation. The measuring of physicochemical parameters, as pH, Redox potential, NH_4_^+^, NO_2_^-^, NO_x_^-^ and organic matter content were conducted by the same method as previously reported ([Cao et al. 2012](#_ENREF_4); [Li et al. 2013](#_ENREF_12)). Dry weight was measured by placing sediments into 105°C oven over night, and water content was calculated accordingly.

According to the physicochemical measurements of these two sediment samples, MaiPo-8 and -9 sediment samples have reduced redox potential states, but more abundant organic matters than that of MaiPo-11 sediment samples. The following parameters are listed:

| **Sample name** | **Sampling position** | **Depth (cm)** | **pH** | **Redox (mV)** | **Water content (%)** | **NH_4_^+^(µm/ kg dry soil)** | **NO_2_^-^ (µm/kg dry soil)** | **NO_2_^-^ +NO_3_^-^ (µm/kg dry soil)** | **Organic matters (%)** |
| --- | --- | --- | --- | --- | --- | --- | --- | --- | --- |
| MaiPo-8 | 22°29.875'N, 114°01.767'E | 10-15 | 7.31 | 298 | 52.4 | 15.96 | 1.75 | 11.19 | 12.9 |
| MaiPo-9 | 22°29.875'N, 114°01.767'E | 20-25 | 7.32 | 241 | 46.4 | 22.41 | 0.50 | 9.10 | 11.1 |
| MaiPo-11 | 22°29.949'N, 114°01.656'E | 13-16 | 7.81 | 170 | 56.3 | 334.54 | 2 | 9.84 | 8.1 |

Sediment samples for metatranscriptomic sequencing were acquired at nearly the same sites and layers with the corresponding samples for metagenome at latter times (Oct 28, 2017 for MaiPo-8, and Jan 7, 2018 for MaiPo-9, -11). After sampling and RNA preservation, samples were immediately transferred to laboratory for RNA isolation and concentration (this step helps to filter rRNA with small molecular weight, such as 5S, 16S rRNA). Transcripts with rRNA filtered out were subjected to metatranscriptomic sequencing.

Total RNA of each sample was extracted using TRIzol Reagent (Invitrogen)/RNeasy Mini Kit (Qiagen). Total RNA of each sample was quantified and qualified by Agilent 2100 Bioanalyzer (Agilent Technologies, Palo Alto, CA, USA), NanoDrop (Thermo Fisher Scientific Inc.) and 1% agrose gel. 1 μg total RNA with RIN value above 7 was used for following library preparation. Next generation sequencing library preparations were constructed according to the manufacturer’s protocol (NEBNext® Ultra™ Directional RNA Library Prep Kit for Illumina®).

The rRNA was removed from total RNA using [Ribo-Zero rRNA Removal Kit (Bacteria)](http://www.illumina.com/products/ribo-zero-rrna-removal-bacteria.html) (Illumina). The ribosomal depleted mRNA was then fragmented and reverse-transcribed. First strand cDNA was synthesized using ProtoScript II Reverse Transcriptase with random primers and Actinomycin D. The second-strand cDNA was synthesized using Second Strand Synthesis Enzyme Mix（include dACG-TP/dUTP）. The purified double-stranded cDNA by AxyPrep Mag PCR Clean-up (Axygen) was then treated with End Prep Enzyme Mix to repair both ends and add a dA-tailing in one reaction, followed by a T-A ligation to add adaptors to both ends. Size selection of Adaptor-ligated DNA was then performed using AxyPrep Mag PCR Clean-up (Axygen), and fragments of ~360 bp (with the approximate insert size of 300 bp) were recovered. The dUTP-marked second strand was digested with Uracil-Specific Excision Reagent (USER) enzyme (New England Biolabs). Each sample was then amplified by PCR for 11 cycles using P5 and P7 primers, with both primers carrying sequences which can anneal with flow cell to perform bridge PCR and P7 primer carrying a six-base index allowing for multiplexing. The PCR products were cleaned up using AxyPrep Mag PCR Clean-up (Axygen), validated using an Agilent 2100 Bioanalyzer (Agilent Technologies, Palo Alto, CA, USA), and quantified by Qubit 2.0 Fluorometer (Invitrogen, Carlsbad, CA, USA).

Then libraries with different barcodes were multiplexed and loaded on an Illumina HiSeq instrument according to manufacturer’s instructions (Illumina, San Diego, CA, USA). Sequencing was carried out using a 2x150 paired-end (PE) configuration; image analysis and base calling were conducted by the HiSeq Control Software (HCS) + OLB + GAPipeline-1.6 (Illumina) on the HiSeq instrument.

**2. Supplementary Note 2: Thermoprofundales diversity based on 16S rRNA genes**

**2.1 IQ tree of Thermoprofundales**


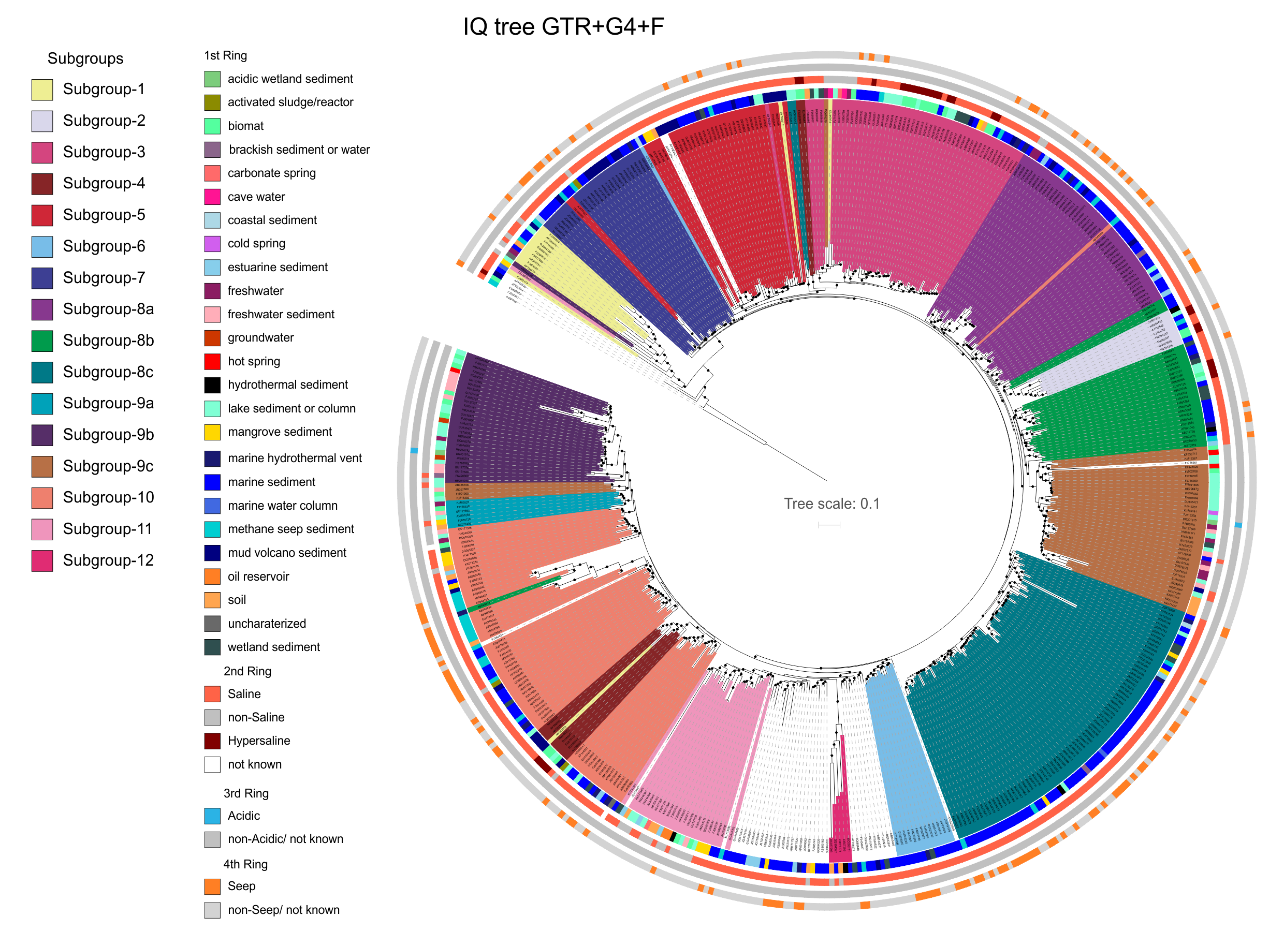
Dereplicated Thermoprofundales sequences (97% cutoff) from SILVA SSURef 128 was used to construct this IQ tree. The 1st ring denotes the environmental categories, the 2nd ring denotes the salinity, and the 3rd ring denotes the acidic condition. Nodes with bootstrap values higher than 75% were marked with black dots. The outgroup was 16S rRNA gene sequence from *Thermoplasma volcanium* GSS1. The subgroups were assigned according to the corresponding RAxML tree (Figure 1a).

**2.2 Phylogenetic tree building method of 16S rRNA gene sequences**

(the tree placing Thermoprofundales archaeon into the Thermoplasmata class)

1. Two random MBG-D sequences from each subgroup (from the result of Figure 1), other representative sequences of lineages in Thermoplasmata, representative sequences of other archaeal groups in Euryarchaeota and an outgroup 16S rRNA gene sequence from Crenarchaeota (*Caldivirga maquilingensis* IC-167, CP000852) were used to construct an arb database ([Ludwig et al. 2004](#_ENREF_13)). All the sequences were aligned by SINA ([Pruesse et al. 2012](#_ENREF_20)).
2. We used 50% MBG-D sequences consensus and ssuref:archaea double filters to export alignment and imported it into CIPRES gateway to get a RAxML BlackBox tree with the default setting (‘-m GTRCAT -f a -N autoMRE’) ([Miller et al. 2010](#_ENREF_16)).
3. We imported the tree back to the arb database, re-curated the sequence names and saved the arb database, exported the tree file, and beautified it with CorelDraw.
4. The highlighted 16S rRNA gene sequences in Figure 3b were from genomic bins (either from SAG or MAG). We assigned their affiliations to individual subgroups according to the ‘SSU_MBG-D.arb’ database by the ARB parsimony quick-add method and also confirmed them in Figure 3b.

**2.3 QIIME scripts to make OTU tables**

filter_samples_from_otu_table.py -i otu_table.biom -o otu_table_over30_OTU.biom -n 30

filter_samples_from_otu_table.py -i otu_table_MBG-D.biom -o otu_table_MBG-D_over10_OTU.biom -n 10

filter_fasta.py -f rep.fna -o rep2.fna -b otu_table_MBG-D_over10_OTU.biom

align_seqs.py -i rep2.fna -t ~/PubDB/QIIME_files/SILVA_128_QIIME_release/core_alignment/core_alignment_SILVA128.fna -o MBG-D_over10_OTU_pynast_aligned/ -p 0.6

filter_alignment.py -i MBG-D_over10_OTU_pynast_aligned/rep2_aligned.fasta -s -g 0.95 -e 0.1 -o MBG-D_over10_OTU_filtered_alignment/

make_phylogeny.py -i MBG-D_over10_OTU_filtered_alignment/rep2_aligned_pfiltered.fasta -o rep2_phylo.tre

beta_diversity_through_plots.py -i otu_table_MBG-D_over10_OTU.biom -m map.txt -o MBG-D_over10_OTU_div_beta/ -t rep2_phylo.tre -e 10

**3. Supplementary Note 3: Co-occurrence network analysis**

**3.1 Network construction**

The ‘over30_OTU table’ (177 studies) was used to make co-occurrence network. The OTU table was filtered by excluding OTUs which have less than 15 sequences among all studies, and less than 6 occurrences among all studies. This step is to screen out underrepresented OTUs of low abundance and limited distribution in few studies and lower the artificial association of OTUs with minor importance in the environments. The network construction step is according to the R scripts offered in the previous publication ([Ju et al. 2014](#_ENREF_9)). Only positive correlations with a spearman’s ρ > 0.4 and Benjamini–Hochberg adjusted *p*-value < 0.01 were considered in the network, which ended up with 205 nodes and 571 edges.

**3.2 C-score calculation**

The checker-board score (C-score) was calculated to test the null model hypothesis of random co-occurrence pattern of network ([Barberán et al. 2011](#_ENREF_2); [Gotelli and McCabe 2002](#_ENREF_8)). The null model was generated by 10,000 times random network stimulation, as:

‘null.model <- oecosimu(matrix, nestedchecker, method = "swap", nsimul=10000)’.

The C-score is an indicator inversely associated with the frequency of co-occurrence. The standardized effect size (SES) was calculated to avid biases of raw C-score value. The positive SES value indicates less co-occurrence comparing to expected by chance. The SES value falls into -2 ~ 2 means the co-occurrence pattern of network is not significantly different from expected by chance.

The resulted SES and C-score of original OTU table and OTU table only containing positive correlation OTUs were 2.113 and 2.786, 160.31 and 134.98 (both *p* value < 0.001), respectively, which means that the observed network is a nonrandom network and has fewer co-occurrence than expected by chance (containing segregated nodes in selective modules).

**3.3 Comparing** **to Erdös–Réyni (ER) random networks**

The identically-sized ER random networks were generated 1,000 times with the same nodes and edges with observed network, and every edge having even probability to be assigned to any node.

The topological property comparison between observed co-occurrence network and ER random network was conducted. The ER random network properties were the average values (and standard deviations) of 1,000 times stimulated identically-sized (205 nodes, 571 edges) ER random networks.

| Network | Clustering coefficient  (CC) | Average path length  (APL) | Modularity (MD) | Graph density (GD) | Network diameter  (ND) | Average degree (AD) |
| --- | --- | --- | --- | --- | --- | --- |
| Observed network | 0.39 | 6.01 | 0.73 | 0.03 | 7.86 | 5.57 |
| ER Random network | 0.03±0.005 | 3.27±0.017 | 0.35±0.015 | 0.03 | 6.36±0.529 | 5.57 |

The observed CC, APL and MD are all greater than their corresponding random ER network values. Additionally, the CC_O_/CC_ER_ of 13 is high, which strongly suggests that the network has “small-world” properties, that is, nodes are more connected than in a identically-sized random network ([Watts and Strogatz 1998](#_ENREF_23)). The MD value higher than 0.4 suggests that the observed network has a modular structure ([Newman 2006](#_ENREF_17)).

**3.4 Degree distribution of the nodes for the observed co-occurrence network and 1,000 ER random network**


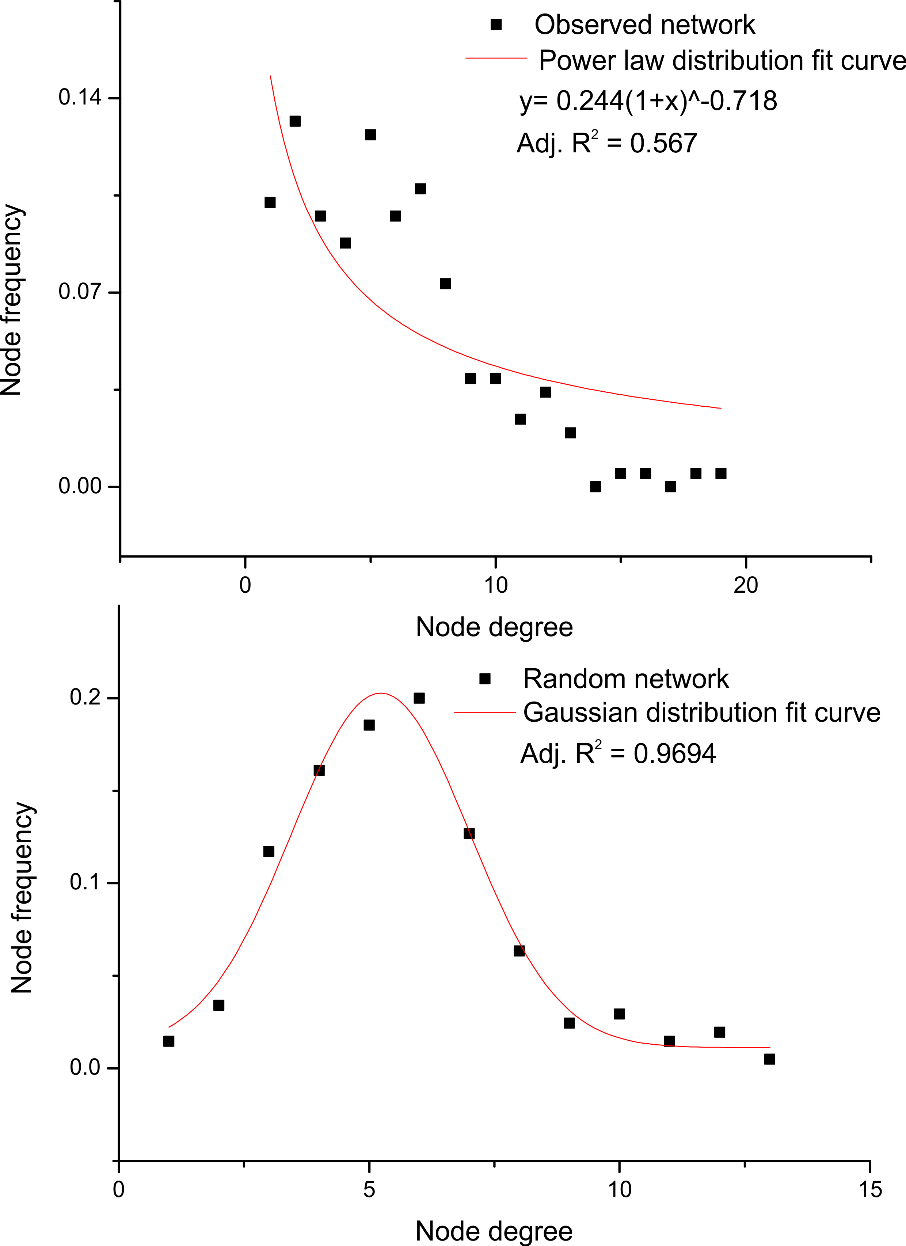


The node degree (the number of edges which are connected to the node) is plotted against the frequency of this node among all nodes in this network. The power law distribution curve is fitted to the observed network, with an equation of y=0.244(1+x)^-0.718^, and adjusted R^2^=0.567, while Gaussian distribution curve fits well to the random network, with adjusted R^2^=0.9694. This indicates that in the observed network, a new node is preferentially attached to nodes with more edges connected, comparing to that in the random network. The scale-free network is defined as a network in which the degree distribution follows a power law, and the fraction of nodes [P(*k*)] in the network having *k* connections to other nodes goes for large values of *k* as P(*k*) ~ *k*^-γ^, in which γ is usually in the range of 2~3 ([Choromański et al. 2013](#_ENREF_5); [Onnela et al. 2007](#_ENREF_19)). Within this observed network, the γ is not in this range. However, this value is compatible with many other ecological, microbial and self-organizing networks ([Steele et al. 2011](#_ENREF_22)), suggesting that this network has meaningful, and nonrandom associations.

**3.5 Observed co-occurring incidence between archaeal lineages**

The observed co-occurring incidence between archaeal groups versus which is stimulated by random association. The observed co-occurring incidence (O) between two archaeal groups was the percentage of observed edge number over the total edge number. The random co-occurring incidence (R) between two archaeal lineages was stimulated by two ways: i) R_ER_ is the mean value of the observed co-occurring incidences for 1,000 identically-sized Erdös-Réyni random networks; ii) R_Theo_ is the theoretical co-occurring incidence calculated by giving the identical frequencies of archaeal lineages and random association between nodes. The co-occurring incidence which contained Thermoprofundales node was highlighted. The O/R ratios which were higher than 1 meant a pattern between two archaeal lineages, that is they were nonrandomly assembled, and indicated a potentially synergetic relationship ([Barberán et al. 2011](#_ENREF_2)).

| Affiliated archaeal group of two nodes | | Frequency of archaeal groups | | Observed edges numbers | The co-occurring incidence (%) | | | | |
| --- | --- | --- | --- | --- | --- | --- | --- | --- | --- |
| Node 1 | Node 2 | Node 1 | Node 2 |  | Observed (O) | Random_ER_ (R_ER_) | Random_Theo_ (R_Theo_) | O/R_ER_ | O/R_Theo_ |
| Bathyarchaeota | Bathyarchaeota | 47 | 47 | 56 | 9.81 | 5.19 | 5.17 | 1.89 | 1.90 |
| Hadesarchaea | Hadesarchaea | 6 | 6 | 3 | 0.53 | 0.21 | 0.07 | 2.46 | 7.32 |
| Lokiarchaeota | Lokiarchaeota | 21 | 21 | 36 | 6.30 | 1.02 | 1.00 | 6.18 | 6.28 |
| Methanomicrobia | Methanomicrobia | 38 | 38 | 87 | 15.24 | 3.35 | 3.36 | 4.54 | 4.53 |
| Thaumarchaeota MG-I | Thaumarchaeota MG-I | 39 | 39 | 92 | 16.11 | 3.52 | 3.54 | 4.58 | 4.55 |
| Thaumarchaeota SCG | Thaumarchaeota SCG | 6 | 6 | 6 | 1.05 | 0.21 | 0.07 | 5.00 | 14.65 |
| **Thermoprofundales** | **Thermoprofundales** | **31** | **31** | **26** | **4.55** | **2.22** | **2.22** | **2.05** | **2.05** |
| Aigarchaeota | Hadesarchaea | 1 | 6 | 1 | 0.18 | 0.20 | 0.03 | 0.89 | 6.10 |
| Aigarchaeota | Thermoplasmatales | 1 | 8 | 1 | 0.18 | 0.21 | 0.04 | 0.85 | 4.58 |
| Bathyarchaeota | Aigarchaeota | 47 | 1 | 1 | 0.18 | 0.28 | 0.22 | 0.62 | 0.78 |
| Bathyarchaeota | Candidate division YNPFFA | 47 | 1 | 4 | 0.70 | 0.34 | 0.22 | 2.08 | 3.12 |
| Bathyarchaeota | Hadesarchaea | 47 | 6 | 9 | 1.58 | 1.36 | 1.35 | 1.16 | 1.17 |
| Bathyarchaeota | Lokiarchaeota | 47 | 21 | 26 | 4.55 | 4.75 | 4.72 | 0.96 | 0.96 |
| Bathyarchaeota | MBG-E | 47 | 3 | 1 | 0.18 | 0.78 | 0.67 | 0.23 | 0.26 |
| Bathyarchaeota | Methanomicrobia | 47 | 38 | 17 | 2.98 | 8.49 | 8.54 | 0.35 | 0.35 |
| Bathyarchaeota | Thaumarchaeota MG-I | 47 | 39 | 25 | 4.38 | 8.72 | 8.77 | 0.50 | 0.50 |
| Bathyarchaeota | Thaumarchaeota SCG | 47 | 6 | 4 | 0.70 | 1.35 | 1.35 | 0.52 | 0.52 |
| Bathyarchaeota | Thermoplasmatales | 47 | 8 | 9 | 1.58 | 1.79 | 1.80 | 0.88 | 0.88 |
| **Bathyarchaeota** | **Thermoprofundales** | **47** | **31** | **29** | **5.08** | **7.00** | **6.97** | **0.73** | **0.73** |
| Candidate division YNPFFA | Methanomicrobia | 1 | 38 | 4 | 0.70 | 0.33 | 0.18 | 2.13 | 3.85 |
| **Candidate division YNPFFA** | **Thermoprofundales** | **1** | **31** | **2** | **0.35** | **0.29** | **0.15** | **1.22** | **2.36** |
| Hadesarchaea | Thermoplasmatales | 6 | 8 | 4 | 0.70 | 0.37 | 0.23 | 1.89 | 3.05 |
| **Hadesarchaea** | **Thermoprofundales** | **6** | **31** | **8** | **1.40** | **0.94** | **0.89** | **1.49** | **1.58** |
| Lokiarchaeota | Hadesarchaea | 21 | 6 | 6 | 1.05 | 0.36 | 0.60 | 2.95 | 1.74 |
| Lokiarchaeota | MHVG | 21 | 2 | 1 | 0.18 | 0.31 | 0.20 | 0.57 | 0.87 |
| Lokiarchaeota | Methanomicrobia | 21 | 38 | 10 | 1.75 | 3.80 | 3.82 | 0.46 | 0.46 |
| Lokiarchaeota | Thermoplasmatales | 21 | 8 | 2 | 0.35 | 0.85 | 0.80 | 0.41 | 0.44 |
| **Lokiarchaeota** | **Thermoprofundales** | **21** | **31** | **36** | **6.30** | **3.10** | **3.11** | **2.03** | **2.03** |
| MBG-E | Hadesarchaea | 3 | 6 | 2 | 0.35 | 0.22 | 0.09 | 1.63 | 4.07 |
| MBG-E | Thaumarchaeota MG-I | 3 | 39 | 5 | 0.88 | 0.60 | 0.56 | 1.45 | 1.56 |
| MBG-E | Thermoplasmatales | 3 | 8 | 2 | 0.35 | 0.24 | 0.11 | 1.48 | 3.05 |
| MHVG | Bathyarchaeota | 2 | 47 | 1 | 0.18 | 0.26 | 0.45 | 0.69 | 0.39 |
| **MHVG** | **Thermoprofundales** | **2** | **31** | **2** | **0.35** | **0.42** | **0.30** | **0.83** | **1.18** |
| Methanobacteria | Bathyarchaeota | 1 | 47 | 1 | 0.18 | 0.24 | 0.22 | 0.74 | 0.78 |
| Methanobacteria | Methanomicrobia | 1 | 38 | 2 | 0.35 | 0.32 | 0.18 | 1.11 | 1.93 |
| **Methanobacteria** | **Thermoprofundales** | **1** | **31** | **2** | **0.35** | **0.29** | **0.15** | **1.19** | **2.36** |
| Methanomicrobia | Hadesarchaea | 38 | 6 | 2 | 0.35 | 0.63 | 1.09 | 0.56 | 0.32 |
| Methanomicrobia | MHVG | 38 | 2 | 1 | 0.18 | 0.33 | 0.36 | 0.53 | 0.48 |
| Methanomicrobia | Thaumarchaeota MG-I | 38 | 39 | 3 | 0.53 | 7.03 | 7.09 | 0.07 | 0.07 |
| **Methanomicrobia** | **Thermoprofundales** | **38** | **31** | **23** | **4.03** | **5.63** | **5.63** | **0.72** | **0.71** |
| Thaumarchaeota MG-I | Thermoplasmatales | 39 | 8 | 2 | 0.35 | 1.49 | 1.49 | 0.24 | 0.23 |
| **Thaumarchaeota MG-I** | **Thermoprofundales** | **39** | **31** | **4** | **0.70** | **5.82** | **5.78** | **0.12** | **0.12** |
| Thaumarchaeota SCG | Methanomicrobia | 6 | 38 | 1 | 0.18 | 0.57 | 1.09 | 0.31 | 0.16 |
| Thaumarchaeota SCG | Thaumarchaeota MG-I | 6 | 39 | 2 | 0.35 | 0.42 | 1.12 | 0.84 | 0.31 |
| Thermoplasmatales | Methanomicrobia | 8 | 38 | 1 | 0.18 | 0.73 | 1.45 | 0.24 | 0.12 |
| **Thermoplasmatales** | **Thermoprofundales** | **8** | **31** | **7** | **1.23** | **1.20** | **1.19** | **1.02** | **1.03** |
| Thermoplasmatales | WSA2 | 8 | 1 | 1 | 0.18 | 0.19 | 0.04 | 0.90 | 4.58 |
| **Thermoprofundales** | **WSA2** | **31** | **1** | **1** | **0.18** | **0.26** | **0.15** | **0.67** | **1.18** |

**4. Supplementary Note 4: Thermoprofundales MAG reconstruction and phylogenomic tree reconstruction**

**4.1 Reference for Thermoprofundales MAG reconstruction**

Besides the MAGs and SAGs which are used in Thermoplasmata 16S rRNA gene tree construction, there are following genomes, MAGs, SAGs and fosmids which were used as the mapping reference in the second step of metagenomic bin construction.

| **Genomes/MAGs/SAGs/fosmids** | **IMG/NCBI accession number/other reference** |
| --- | --- |
| Deep sea and seafloor MG-II/-III metagenomic bins | ([Li et al. 2015](#_ENREF_11)) |
| Deep-Mediterranean MG-II/-III fosmids | ([Deschamps et al. 2014](#_ENREF_7)) |
| Methanogenic archaeon ISO4-H5 | 2660238307 |
| SCGC AAA007-O11 (MG-III) | AQTW01000000 |
| SCGC AAA288-C18 (MG-II) | ARPJ00000000 |
| SCGC AAA288-E19 (MG-III) | AQTX00000000 |
| SCGC AB-629-J06 (MG-II) | AQVM00000000 |
| *Thermoplasma volcanium* GSS1 | NC_002689 |
| Uncultured marine group II Euryarchaeote fosmids | ([Martin-Cuadrado et al. 2008](#_ENREF_15)) |
| Uncultured marine group III Euryarchaeote fosmids | ([Martin-Cuadrado et al. 2008](#_ENREF_15)) |
| Pearl River estuary water column metagenomic bin MGII-CZ | Unpublished data |
| Maipo_8_all_bin.033 | 1 step Thermoplasmata bins (Maipo-8) |
| Maipo_8_all_bin.060 | 1 step Thermoplasmata bins (Maipo-8) |
| Maipo_8_all_bin.096 | 1 step Thermoplasmata bins (Maipo-8) |
| Maipo_8_all_bin.442 | 1 step Thermoplasmata bins (Maipo-8) |
| Maipo_8_all_bin.464 | 1 step Thermoplasmata bins (Maipo-8) |
| Maipo_8_all_bin.565 | 1 step Thermoplasmata bins (Maipo-8) |
| Maipo-9_Maxbin_for_original_1k.027 | 1 step Thermoplasmata bins (Maipo-9) |
| Maipo-9_Maxbin_for_original_1k.214 | 1 step Thermoplasmata bins (Maipo-9) |
| Maipo-9_Maxbin_for_original_1k.006 | 1 step Thermoplasmata bins (Maipo-9) |
| Maipo-9_Maxbin_for_original_1k.047 | 1 step Thermoplasmata bins (Maipo-9) |
| Maipo-9_Maxbin_for_original_1k.438 | 1 step Thermoplasmata bins (Maipo-9) |
| Maipo-9_Maxbin_for_original_1k.016 | 1 step Thermoplasmata bins (Maipo-9) |
| Maipo-9_Maxbin_for_original_1k.210 | 1 step Thermoplasmata bins (Maipo-9) |
| Maipo-9_Maxbin_for_original_1k.454 | 1 step Thermoplasmata bins (Maipo-9) |
| Maipo_11_all_bin.203 | 1 step Thermoplasmata bins (Maipo-11) |
| Maipo_11_all_bin.288 | 1 step Thermoplasmata bins (Maipo-11) |
| Maipo_11_all_bin.321 | 1 step Thermoplasmata bins (Maipo-11) |
| Maipo_11_all_bin.454 | 1 step Thermoplasmata bins (Maipo-11) |
| Maipo_11_all_bin.094 | 1 step Thermoplasmata bins (Maipo-11) |
| Maipo_11_all_bin.223 | 1 step Thermoplasmata bins (Maipo-11) |
| Maipo_11_all_bin.296 | 1 step Thermoplasmata bins (Maipo-11) |
| Maipo_11_all_bin.431 | 1 step Thermoplasmata bins (Maipo-11) |
| Maipo_11_all_bin.168 | 1 step Thermoplasmata bins (Maipo-11) |
| Maipo_11_all_bin.287 | 1 step Thermoplasmata bins (Maipo-11) |
| Maipo_11_all_bin.305 | 1 step Thermoplasmata bins (Maipo-11) |
| Maipo_11_all_bin.449 | 1 step Thermoplasmata bins (Maipo-11) |

**4.2 MAGs and SAGs included in the phylogenetic tree of concatenated 43 marker genes (Figure 3a)**

| **Genomes/MAGs/SAGs** | **IMG/NCBI accession number** |
| --- | --- |
| *Candidatus* Methanomassiliicoccus intestinalis Issoire-Mx1 | NC_021353 |
| *Candidatus* Methanomethylophilus alvus Mx1201 | NC_020913 |
| *Candidatus* Methanoplasma termitum Mpt1 | IMG: 2636415782 |
| Methanomassiliicoccales archaeon RumEn M1 | IMG: 2667527221 |
| Methanomassiliicoccales archaeon RumEn M2 | IMG: 2667527222 |
| SCGC AB-539-C06 | AOSH01000000 |
| SCGC AB-539-N05 | ALXL01000000 |
| SCGC AB-540-F20 | AOSI01000000 |
| *Thermoplasma acidophilum* DSM 1728 | AL139299 |
| Thermoplasmatales archaeon BRNA1 | CP002916 |
| uncultured marine group II euryarchaeote | CM001443 |
| Uncultured *Thermoplasmata* T3F75 | IMG: 2571042356 |
| Uncultured *Thermoplasmata* T3M75 | IMG: 2571042357 |
| *Methanomassiliicoccus luminyensis* B10 | NZ_CAJE01000026 |
| Thermoplasmatales archaeon Gp1 | ATDV01000004 |
| Euryarchaeota archaeon SG8-5 | LSSH00000000 |
| SCGC AAA288-E19 | AQTX00000000 |
| M8B2D | This study |
| M9B1D | This study |
| M9B2D | This study |
| 3300003432B1D | IMG: 3300003432 (Binned in this study) |
| Thermoplasmatales archaeon SM1-50 | LSSI00000000 |
| M11B2D | This study |

**5. Supplementary Note 5: Thermoprofundales MAG metabolism analysis**

**5.1 Gene presence/absence LEfSe analysis**

For the gene presence and absence table, we used the LDA Effect Size analysis (LEfSe) ([Segata et al. 2011](#_ENREF_21)) to investigate the genes that are significantly associated with certain Thermoprofundales subgroups. In this analysis, the LEfSe analysis uses non-parametric factorial Kruskal-Wallis (KW) sum-rank test to detect features with significant differential abundance with respect to the class of interest (the Thermoprofundales subgroups); then it will further use Linear Discriminant Analysis to estimate the effect size of each differentially abundant feature. The outcome is represented in the following figure.


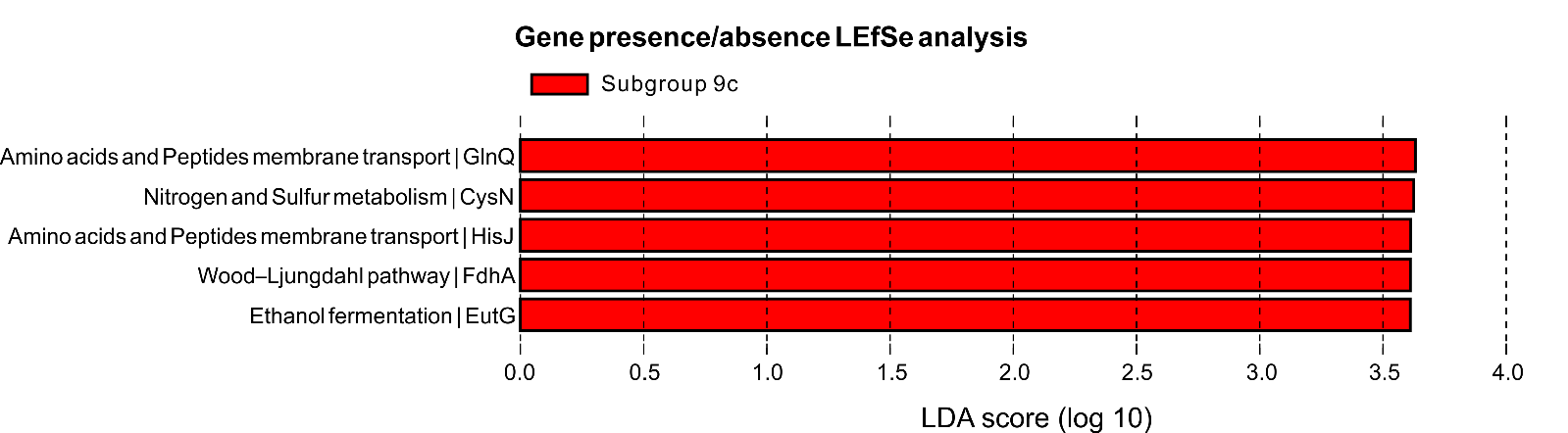


Plot of LEfSe results. The genes that are of significant differential distribution in the Thermoprofundales subgroups were indicated by the LEfSe analysis. The LEfSe analyses were performed using default settings, with per-sample normalization of the sum of the values to 1M in the data formatting step and “All-against-all” (more strict) strategy for multi-class analysis in the LEfSe analyzing step. Only the hits of discriminative features with abs LDA score > 2.0 were depicted. Five genes were indicated to be significantly associated with Thermoprofundales Subgroup 9c out of 110 featured metabolic genes.

**5.2 Metabolism comparison among genomes of Thermoplasmata**

The coexistence of H_4_folate-WL pathway and MtrA-H complex were not discovered in the reference Thermoplasmata genomes that were deposited in the KEGG Organisms database (https://www.genome.jp/kegg/kegg2.html).

| **Genomes** | **H_4_folate-WL pathway** | **H_4_MPT-WL pathway** | **MtrA-H complex** | **McrABC** |
| --- | --- | --- | --- | --- |
| Thermoplasmatales; Thermoplasmataceae; Thermoplasma acidophilum DSM 1728 | Yes | No | No | No |
| Thermoplasmatales; Thermoplasmataceae; Thermoplasma volcanium GSS1 | Yes | No | No | No |
| Thermoplasmatales; Picrophilaceae; Picrophilus torridus DSM 9790 | Yes | No | No | No |
| Thermoplasmatales; Ferroplasmaceae; Ferroplasma acidarmanus fer1 | Yes | No | No | No |
| Thermoplasmatales; Ferroplasmaceae; Ferroplasma acidiphilum Y | Yes | No | No | No |
| Thermoplasmatales; Cuniculiplasmataceae; Cuniculiplasma divulgatum PM4 | Yes | No | No | No |
| Thermoplasmatales archaeon BRNA1 | Yes | No | No | Yes |
| Methanomassiliicoccales; Methanomassiliicoccaceae; Candidatus Methanomethylophilus alvus Mx1201 | Yes | No | No | Yes |
| Methanomassiliicoccales; Methanomassiliicoccaceae; Methanomassiliicoccus intestinalis Issoire-Mx1 | Yes | Yes | No | Yes |
| Methanomassiliicoccales; Methanomassiliicoccaceae; Candidatus Methanoplasma termitum MpT1 | Yes | Yes | No | Yes |
| unclassified Thermoplasmata; Methanogenic archaeon ISO4-H5 | Yes | No | No | Yes |

| **Archaeal group** | **Genome name** | **codhD (delta)** | **codhC (beta)** | **codh (catalytic)** | **Wood**–**Ljungdahl pathway (markers)** |
| --- | --- | --- | --- | --- | --- |
|  |  | **TIGR00381** | **TIGR00316** | **TIGR01702** |  |
|  | *Thermoplasmatales* |  |  |  |  |
| Thermoplasmatales | Archaea_Euryarchaeota_Thermoplasmata_Acidiplasma_aeolicum | 0 | 0 | 0 | 0 |
| Thermoplasmatales | Archaea_Euryarchaeota_Thermoplasmata_Cuniculiplasma_divulgatum | 0 | 0 | 0 | 0 |
| Thermoplasmatales | Archaea_Euryarchaeota_Thermoplasmata_Picrophilus_torridus_DSM_9790 | 0 | 0 | 0 | 0 |
| Thermoplasmatales | Archaea_Euryarchaeota_Thermoplasmata_Thermoplasma_volcanium_GSS1 | 0 | 0 | 0 | 0 |
| Thermoplasmatales | Archaea_Euryarchaeota_Thermoplasmata_Thermoplasmatales_archaeon_I-plasma | 0 | 0 | 0 | 0 |
|  | *Marine Group II* |  |  |  |  |
| Marine Group II | Archaea_Euryarchaeota_unclassified_Euryarchaeota_Marine_group_II_euryarchaeote_REDSEA-S11_B3N4 | 0 | 0 | 0 | 0 |
| Marine Group II | Archaea_Euryarchaeota_unclassified_Euryarchaeota_Marine_group_II_Guaymas21 | 0 | 0 | 0 | 0 |
| Marine Group II | Archaea_Euryarchaeota_unclassified_Euryarchaeota_Marine_group_II_Guaymas25 | 0 | 0 | 0 | 0 |
| Marine Group II | Archaea_Euryarchaeota_unclassified_Euryarchaeota_Marine_group_II_Guaymas28 | 0 | 0 | 0 | 0 |
| Marine Group II | Archaea_Euryarchaeota_unclassified_Euryarchaeota_uncultured_marine_group_II_euryarchaeote | 0 | 0 | 0 | 0 |
|  | *Marine Group III* |  |  |  |  |
| Marine Group III | Archaea_Euryarchaeota_unclassified_Euryarchaeota_Marine_Group_III_euryarchaeote_CG-Bathy1 | 0 | 0 | 0 | 0 |
| Marine Group III | Archaea_Euryarchaeota_unclassified_Euryarchaeota_Marine_Group_III_euryarchaeote_CG-Epi1 | 0 | 0 | 0 | 0 |
| Marine Group III | Archaea_Euryarchaeota_unclassified_Euryarchaeota_Marine_Group_III_euryarchaeote_CG-Epi2 | 0 | 0 | 0 | 0 |
| Marine Group III | Archaea_Euryarchaeota_unclassified_Euryarchaeota_Marine_group_III_Guaymas31 | 0 | 0 | 0 | 0 |
|  | *Methanomassiliicoccales* |  |  |  |  |
| Methanomassiliicoccales | Archaea_Euryarchaeota_Thermoplasmata_Candidatus_Methanomassiliicoccus_intestinalis_Issoire-Mx1 | 0 | 1 | 0 | 1 |
| Methanomassiliicoccales | Archaea_Euryarchaeota_Thermoplasmata_Candidatus_Methanomethylophilus_alvus_Mx1201 | 0 | 0 | 0 | 0 |
| Methanomassiliicoccales | Archaea_Euryarchaeota_Thermoplasmata_Candidatus_Methanoplasma_termitum | 0 | 0 | 1 | 1 |
| Methanomassiliicoccales | Archaea_Euryarchaeota_Thermoplasmata_Methanomassiliicoccales_archaeon_RumEn_M1 | 0 | 1 | 1 | 1 |
| Methanomassiliicoccales | Archaea_Euryarchaeota_Thermoplasmata_Methanomassiliicoccus_luminyensis_B10 | 1 | 0 | 0 | 1 |

**5.3 Metabolism comparison of the makers of Wood–Ljungdahl pathway among genomes of Thermoplasmata**

The assignment of the markers was performed by scanning the genomes to TIGRfam database with the suggested cutoff settings. Details were similar to those described in the reference ([Anantharaman et al. 2016](#_ENREF_1)).

**5.4 Phylogenetic tree of Thermoprofundales AdhP and EutG related proteins**


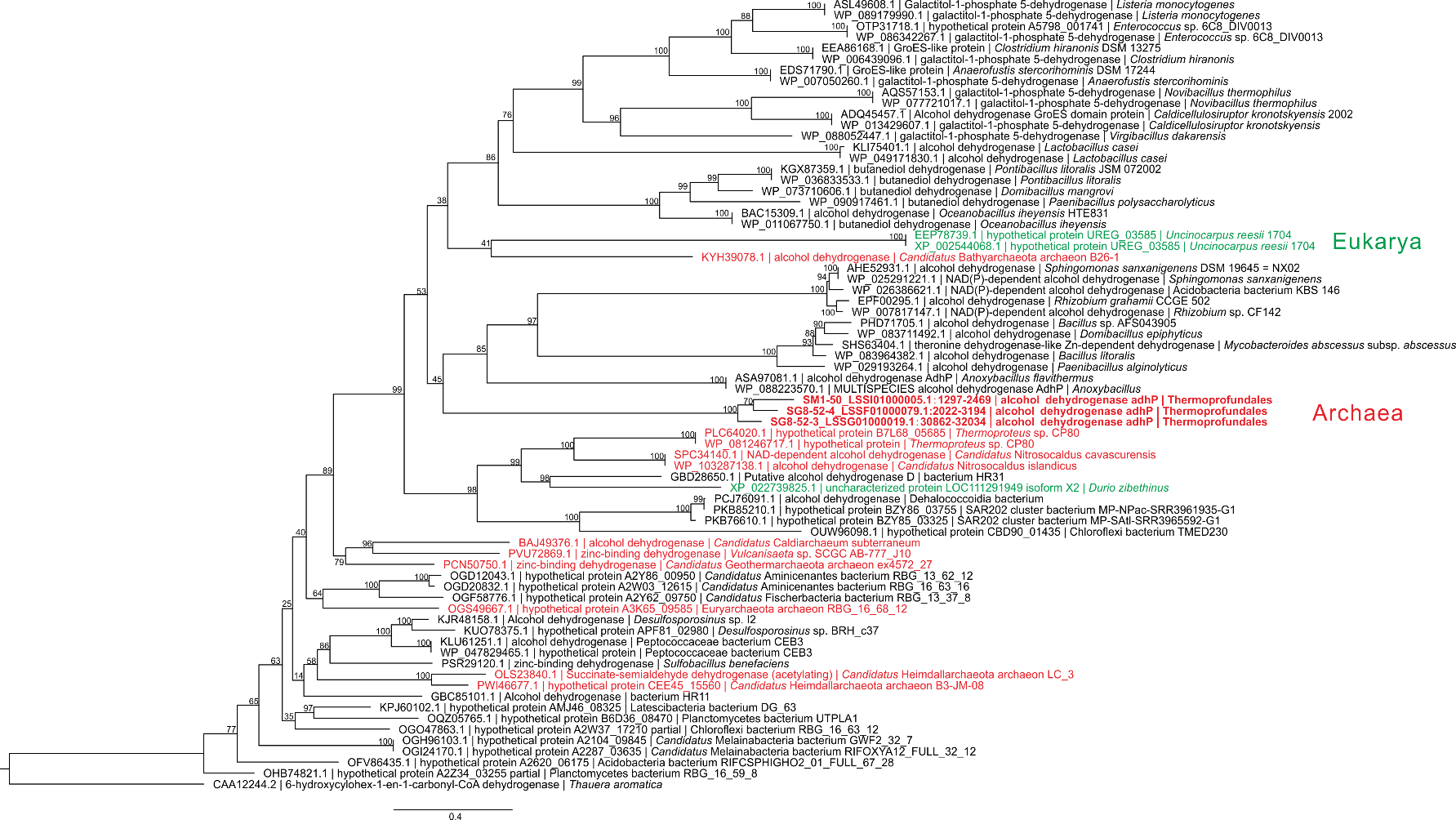


Phylogeny of Thermoprofundales AdhP related proteins. The protein alignment contained proteins from the best hits of Thermoprofundales AdhP (MAG: SM1-50; LSSI01000005.1:1297-2469) by BLASTP in NCBI, and was aligned by MAFFT ([Katoh and Standley 2014](#_ENREF_10)). We used 1 or 2 hits from the same taxonomy group. This tree was constructed by IQ -TREE ([Nguyen et al. 2014](#_ENREF_18)) by “-m MFP -mset LG,WAG -mrate E,I,G,I+G -mfreq FU -bb 1000”. The resulted ultrafast bootstrap values are relatively higher than the corresponding default RAxML bootstrap values. The Thermoprofundales AdhP sequences are labeled bold. Archaeal sequences are labeled red and eukaryotic sequences are labeled green. The tree was rooted by a 6-hydroxycyclohex-1-ene-1-carbonyl-CoA dehydrogenase that contained the non-specific domain hit (dearomat_had) to the Thermoprofundales AdhP in NCBI-CDD database ([Marchler-Bauer et al. 2014](#_ENREF_14)).


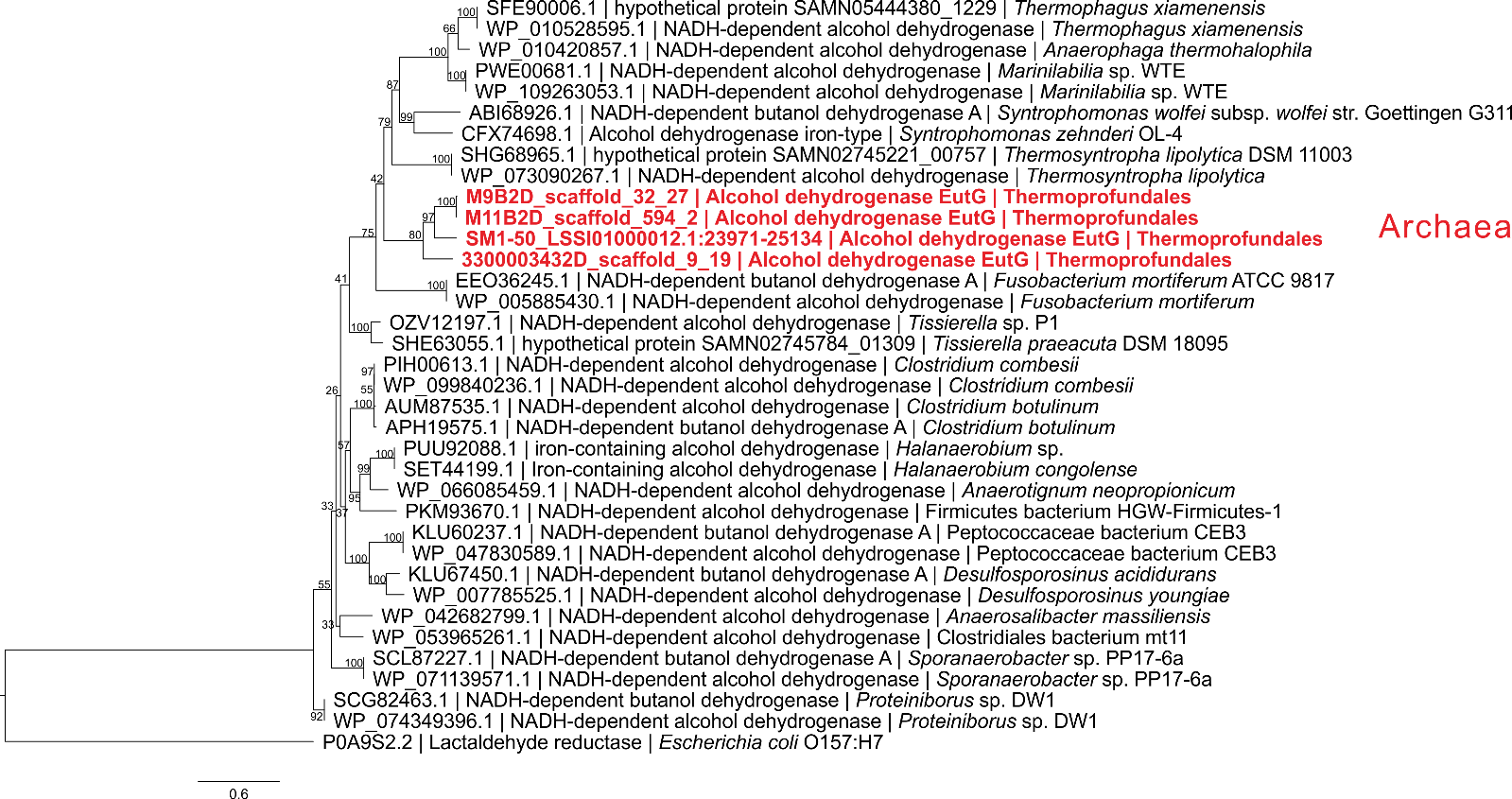
Phylogeny of Thermoprofundales EutG related proteins. The protein alignment contained proteins from the best hits of Thermoprofundales EutG (MAG: SM1-50; LSSI01000012.1:23971-25134) by BLASTP in NCBI, and was aligned by MAFFT ([Katoh and Standley 2014](#_ENREF_10)). We used 1 or 2 hits from the same taxonomy group. This tree was constructed by IQ -TREE ([Nguyen et al. 2014](#_ENREF_18)) using “-m MFP -mset LG,WAG -mrate E,I,G,I+G -mfreq FU -bb 1000” setting. The resulted ultrafast bootstrap values are relatively higher than the corresponding default RAxML bootstrap values. The Thermoprofundales EutG sequences are labeled bold. Archaeal sequences are labeled red. The tree was rooted by a lactaldehyde reductase that contained the non-specific domain hit (lactal_redase) to the Thermoprofundales EutG in NCBI-CDD database ([Marchler-Bauer et al. 2014](#_ENREF_14)).

| **Protein** | **PROSITE hit region** | **Hit sequence** | **Motif** | **Description** |
| --- | --- | --- | --- | --- |
| ‘Thermoplasmatales' archaeon SM1-50 AdhP (LSSI01000005.1:1297-2469) | 58-72 | GHEiSGIvkevGksA | [PS00059](https://prosite.expasy.org/cgi-bin/prosite/nicedoc.pl?PS00059) | ADH_ZINC   Zinc-containing alcohol dehydrogenases signature |
| ‘Thermoplasmatales' archaeon SG8-52-3 AdhP (LSSG01000019.1:30862-32034) | 58-72 | GHEiAGIveeiGksS | [PS00059](https://prosite.expasy.org/cgi-bin/prosite/nicedoc.pl?PS00059) | ADH_ZINC   Zinc-containing alcohol dehydrogenases signature |
| ‘Thermoplasmatales' archaeon SG8-52-4 AdhP (LSSF01000079.1:2022-3194) | 58-72 | GHEiAGVveklGksA |  |  |
|  |  |  |  |  |
| ‘Thermoplasmatales' archaeon SM1-50 EutG (LSSI01000012.1:23971-25134) | 265-285 | WaTHgiEHaVSAaydViHGvG | PS00060 | ADH_IRON_2   Iron-containing alcohol dehydrogenases signature 2 |
| Thermoprofundales 3300003432D (scaffold_9_19) | 265-285 | WaTHgmEHaLSAtydVtHGvG | PS00060 | ADH_IRON_2   Iron-containing alcohol dehydrogenases signature 2 |
| Thermoprofundales M9B2D (scaffold_32_27) | 168-196 | SIlDptytftvPphhsAaGivDIFshilE | PS00913 | ADH_IRON_1   Iron-containing alcohol dehydrogenases signature 1 |
| Thermoprofundales M9B2D (scaffold_32_27) | 259-279 | WaTHaiEHaVSAtydVtHGvG | PS00060 | ADH_IRON_2   Iron-containing alcohol dehydrogenases signature 2 |
| Thermoprofundales M11B2D (scaffold_594_2) | 168-196 | SIlDptytftvPphhsAaGivDIFshilE | PS00913 | ADH_IRON_1   Iron-containing alcohol dehydrogenases signature 1 |
| Thermoprofundales M11B2D (scaffold_594_2) | 259-279 | WaTHaiEHaVSAtydVtHGvG | PS00060 | ADH_IRON_2   Iron-containing alcohol dehydrogenases signature 2 |

**5.5 The functional sites and conserved domains of Thermoprofundales AdhP and EutG proteins**

The functional sites were identified by ScanProsite with “2018_05” release using the default settings ([De Castro et al. 2006](#_ENREF_6)). The old names of ‘Thermoplasmatales’ archaeon are used in the table, but they are in fact affiliated to Thermoprofundales as figured out in this study.

| **Query** | **Hit type** | **PSSM-ID** | **From** | **To** | **E-Value** | **Bitscore** | **Accession** | **Domain Name** | **Incomplete** | **Superfamily** | **Superfamily Name** |
| --- | --- | --- | --- | --- | --- | --- | --- | --- | --- | --- | --- |
| ‘Thermoplasmatales' archaeon SM1-50 EutG (LSSI01000012.1:23971-25134) | specific | 173946 | 3 | 384 | 0 | 583.26 | cd08187 | BDH (Butanol dehydrogenase) | - | cl02872 | iron-containing alcohol dehydrogenase superfamily |
| Thermoprofundales 3300003432D (scaffold_9_19) | specific | 173946 | 3 | 384 | 0 | 604.061 | cd08187 | BDH (Butanol dehydrogenase) | - | cl02872 | iron-containing alcohol dehydrogenase superfamily |
| Thermoprofundales M9B2D (scaffold_32_27) | specific | 173946 | 3 | 378 | 0 | 604.061 | cd08187 | BDH (Butanol dehydrogenase) | - | cl02872 | iron-containing alcohol dehydrogenase superfamily |
| Thermoprofundales M11B2D (scaffold_594_2) | specific | 173946 | 3 | 378 | 0 | 605.216 | cd08187 | BDH (Butanol dehydrogenase) | - | cl02872 | iron-containing alcohol dehydrogenase superfamily |
|  | | | | | | | | | | | |
| **Query** | **Hit type** | **PSSM-ID** | **From** | **To** | **E-Value** | **Bitscore** | **Accession** | **Domain Name** | **Incomplete** | **Superfamily** | **Superfamily Name** |
| ‘Thermoplasmatales' archaeon SM1-50 AdhP (LSSI01000005.1:1297-2469) | superfamily | 327354 | 1 | 376 | 1.62E-54 | 183.197 | cl16912 | - | - | MDR superfamily | Medium chain reductase/dehydrogenase (MDR)/zinc-dependent alcohol dehydrogenase-like family |
| ‘Thermoplasmatales' archaeon SG8-52-3 AdhP (LSSG01000019.1:30862-32034) | superfamily | 327354 | 1 | 385 | 1.73E-59 | 195.928 | cl16912 | - | - | MDR superfamily | Medium chain reductase/dehydrogenase (MDR)/zinc-dependent alcohol dehydrogenase-like family |
| ‘Thermoplasmatales' archaeon SG8-52-4 AdhP (LSSF01000079.1:2022-3194) | specific | 223992 | 1 | 389 | 6.38E-58 | 192.032 | COG1064 | (AdhP) D-arabinose 1-dehydrogenase, Zn-dependent alcohol dehydrogenase family | - | cl25577 | Enoylreductase in Polyketide synthases |

The conserved domain and superfamily hits were identified by Web CD-Search Tool in NCBI-CDD database using the default settings ([Marchler-Bauer et al. 2014](#_ENREF_14)). The old names of ‘Thermoplasmatales’ archaeon are used in the table, but they are in fact affiliated to Thermoprofundales as figured out in this study.

References:

Anantharaman K, Brown CT, Hug LA, Sharon I, Castelle CJ, Probst AJ, Thomas BC, Singh A, Wilkins MJ, Karaoz U, Brodie EL, Williams KH, Hubbard SS, Banfield JF (2016) Thousands of microbial genomes shed light on interconnected biogeochemical processes in an aquifer system. Nat Commun 7:13219 doi:10.1038/ncomms13219

Barberán A, Bates ST, Casamayor EO, Fierer N (2011) Using network analysis to explore co-occurrence patterns in soil microbial communities. ISME J 6:343 doi:10.1038/ismej.2011.119

Bushnell B (2014) BBMap: A Fast, Accurate, Splice-Aware Aligner. Paper presented at the The 9th Annual Genomics of Energy & Environment Meeting, Walnut Creek, CA, March 17-20, 2014

Cao H, Hong Y, Li M, Gu J-D (2012) Community shift of ammonia-oxidizing bacteria along an anthropogenic pollution gradient from the Pearl River Delta to the South China Sea. Appl Microbiol Biotechnol 94(1):247-259 doi:10.1007/s00253-011-3636-1

Choromański K, Matuszak M, Miȩkisz J (2013) Scale-free graph with preferential attachment and evolving internal vertex structure. Journal of Statistical Physics 151(6):1175-1183

De Castro E, Sigrist CJ, Gattiker A, Bulliard V, Langendijk-Genevaux PS, Gasteiger E, Bairoch A, Hulo N (2006) ScanProsite: detection of PROSITE signature matches and ProRule-associated functional and structural residues in proteins. Nucleic Acids Res 34(suppl_2):W362-W365 doi:10.1093/nar/gkl124

Deschamps P, Zivanovic Y, Moreira D, Rodriguez-Valera F, López-García P (2014) Pangenome evidence for extensive interdomain horizontal transfer affecting lineage core and shell genes in uncultured planktonic thaumarchaeota and euryarchaeota. Genome Biol Evol 6(7):1549-1563

Gotelli NJ, McCabe DJ (2002) Species co‐occurrence: A meta‐analysis of jm diamond's assembly rules model. Ecology 83(8):2091-2096

Ju F, Xia Y, Guo F, Wang Z, Zhang T (2014) Taxonomic relatedness shapes bacterial assembly in activated sludge of globally distributed wastewater treatment plants. Environ Microbiol 16(8):2421-2432 doi:10.1111/1462-2920.12355

Katoh K, Standley DM (2014) MAFFT: iterative refinement and additional methods. Multiple Sequence Alignment Methods:131-146

Li M, Baker BJ, Anantharaman K, Jain S, Breier JA, Dick GJ (2015) Genomic and transcriptomic evidence for scavenging of diverse organic compounds by widespread deep-sea archaea. Nat Commun 6:8933 doi:10.1038/ncomms9933

Li M, Hong Y, Cao H, Gu J-D (2013) Community structures and distribution of anaerobic ammonium oxidizing and *nirS*-encoding nitrite-reducing bacteria in surface sediments of the South China Sea. Microb Ecol 66(2):281-96 doi:10.1007/s00248-012-0175-y

Ludwig W, Strunk O, Westram R, Richter L, Meier H, Yadhukumar, Buchner A, Lai T, Steppi S, Jobb G, Forster W, Brettske I, Gerber S, Ginhart AW, Gross O, Grumann S, Hermann S, Jost R, Konig A, Liss T, Lussmann R, May M, Nonhoff B, Reichel B, Strehlow R, Stamatakis A, Stuckmann N, Vilbig A, Lenke M, Ludwig T, Bode A, Schleifer KH (2004) ARB: a software environment for sequence data. Nucleic Acids Res 32(4):1363-1371 doi:10.1093/nar/gkh293

Marchler-Bauer A, Derbyshire MK, Gonzales NR, Lu S, Chitsaz F, Geer LY, Geer RC, He J, Gwadz M, Hurwitz DI (2014) CDD: NCBI's conserved domain database. Nucleic Acids Res 43(D1):D222-D226 doi:10.1093/nar/gku1221

Martin-Cuadrado A-B, Rodriguez-Valera F, Moreira D, Alba JC, Ivars-Martínez E, Henn MR, Talla E, López-García P (2008) Hindsight in the relative abundance, metabolic potential and genome dynamics of uncultivated marine archaea from comparative metagenomic analyses of bathypelagic plankton of different oceanic regions. ISME J 2(8):865-886

Miller MA, Pfeiffer W, Schwartz T (2010) Creating the CIPRES Science Gateway for inference of large phylogenetic trees. Paper presented at the Gateway Computing Environments Workshop (GCE), 2010, New Orleans, Louisiana, USA,

Newman ME (2006) Modularity and community structure in networks. Proc Natl Acad Sci U S A 103(23):8577-8582

Nguyen L-T, Schmidt HA, von Haeseler A, Minh BQ (2014) IQ-TREE: a fast and effective stochastic algorithm for estimating maximum-likelihood phylogenies. Mol Biol Evol 32(1):268-274

Onnela J-P, Saramäki J, Hyvönen J, Szabó G, Lazer D, Kaski K, Kertész J, Barabási A-L (2007) Structure and tie strengths in mobile communication networks. Proc Natl Acad Sci U S A 104(18):7332-7336

Pruesse E, Peplies J, Gloeckner FO (2012) SINA: Accurate high-throughput multiple sequence alignment of ribosomal RNA genes. Bioinformatics 28(14):1823-1829 doi:10.1093/bioinformatics/bts252

Segata N, Izard J, Waldron L, Gevers D, Miropolsky L, Garrett WS, Huttenhower C (2011) Metagenomic biomarker discovery and explanation. Genome Biol 12(6):R60 doi:10.1186/gb-2011-12-6-r60

Steele JA, Countway PD, Xia L, Vigil PD, Beman JM, Kim DY, Chow C-ET, Sachdeva R, Jones AC, Schwalbach MS (2011) Marine bacterial, archaeal and protistan association networks reveal ecological linkages. ISME J 5(9):1414-1425

Watts DJ, Strogatz SH (1998) Collective dynamics of ‘small-world’networks. Nature 393(6684):440-442

**
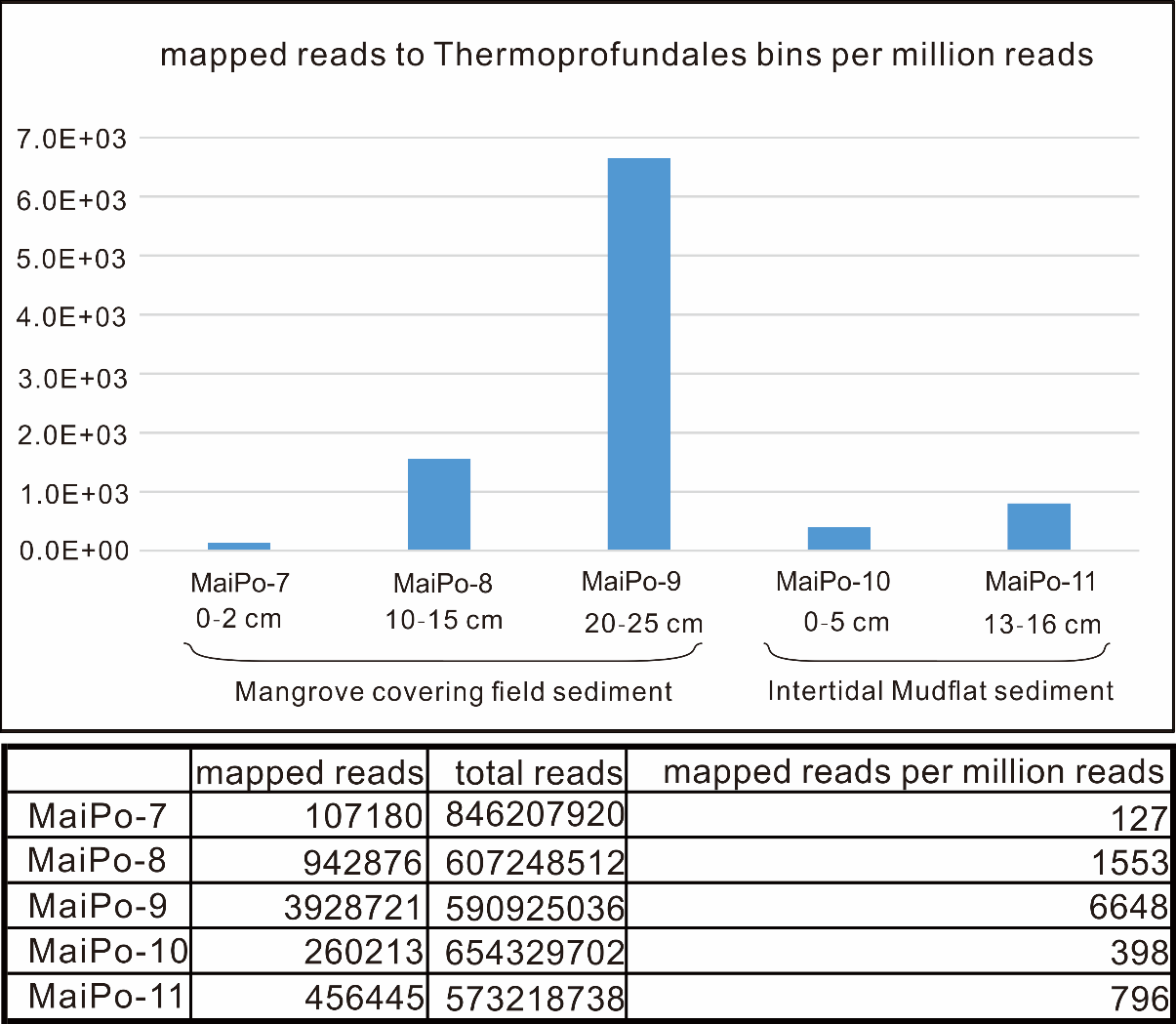
Supplementary Figures and Tables**

**Figure S1. The abundance ranking diagram indicating mapped raw reads per normalized genome size of Thermoprofundales MAGs in each metagenome data.** The marker gene set for genomic binning is 40 and minimum scaffold length is 1kb for MaxBin, respectively. Mapping was done by BBmap with the default setting. The MaiPo-7 is the surface sediment sample in the sediment core of MaiPo-8 and 9, as depicted in the figure; The MaiPo-10 is the surface sediment sample in the sediment core of MaiPo-11. Their detailed information and metagenomes are deposited in DOE JGI-IMG (MaiPo-7: 3300022562 and MaiPo-10: 3300010412).

**
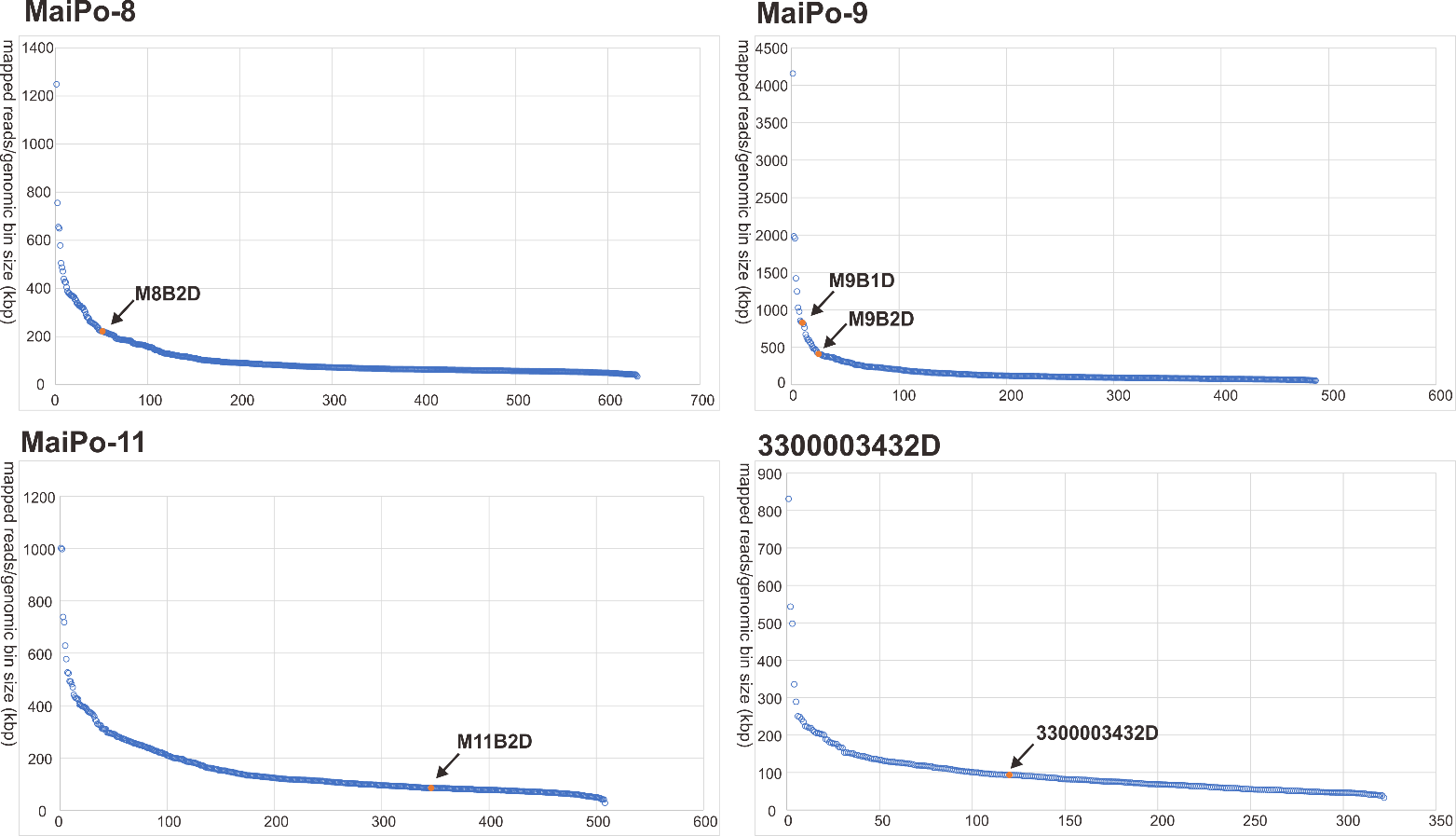
Figure S2. Mapped raw read numbers to Thermoprofundales bins per million reads of metagenomes from Mai Po wetland sediment samples.** The x-axis stands for the sorted rank of genomic bins that are constructed from the corresponding metagenome. The y-axis stands for the mapped raw read number [normalized by genomic bin size (kbp)] of each genomic bin in the corresponding metagenome (normalized by million reads). The figure reflects the relative abundance of genomic bins in their corresponding metagenomes. BBmap was applied with the default setting for mapping reads ([Bushnell 2014](#_ENREF_3)). MaiPo-7 and MaiPo-10 were the corresponding surface layer sediment samples in the mangrove covering fields and intertidal mudflats. The Thermoprofundales bins are labeled red in each subfigure.

**Table S1. MBG-D Sequence information from SILVA SSU 128 database (8503 sequences, Sheet1) and the statistics of sequence counts from different environment categories (Sheet2)**

**Table S2. Species abundance distribution (SAD) table representing occurrence and abundance pattern of archaeal groups in all the studies.** This table is based on ‘over30_OTU table’ (each study contains at least 30 archaeal sequences). Overall, there are 177 studies that are included in the SAD analysis.

**Table S3. The summary of intracellular and extracellular peptidases from eight Thermoprofundales MAGs**. The MERPOS family IDs were assigned by BLAST to MERPOS database, and duplicated annotations were screened out manually. The MERPOS families which distributed in more than 75% of the eight Thermoprofundales bins were labeled in grey. The extracellular peptidases were identified by predicting the transport signal peptidases using POSRTb and PRED-SIGNAL softwares, and only congruent results from both of them led to assigning an extracellular peptidase. The MERPOS predicted peptidases were also compared to annotations by nr and Pfam database.

**Table S4. Annotation of eight Thermoprofundales bins by nr database, BlastKOALA, and EggNOG 4.5.1**. The nr annotation and the corresponding taxonomy information were retrieved from the non-hypothetic protein top-hit. The top arNOG hit was assigned by EggNOG 4.5.1 database using HMM mapping mode. The resulted arCOG numbers were used to search against arCOG database (ftp://ftp.ncbi.nih.gov/pub/koonin/arCOGs) to retrieve their annotations in detail. The protein encoded genes for the major pathways and metabolisms in Figure 2 were listed in Sheet1-Sheet13, and the rest were listed in Sheet14.

**Table S5. The expressing level of proteins of MAGs from this study based on transcriptomic analysis.** Proteins in bold are those that participate in major pathways (Figure 4) or that are peptidases (Table S3).
